# Production of recombinant vesicular stomatitis virus-based vectors by tangential flow depth filtration

**DOI:** 10.1007/s00253-024-13078-6

**Published:** 2024-02-27

**Authors:** Sven Göbel, Lars Pelz, Cristina A. T. Silva, Béla Brühlmann, Charles Hill, Jennifer Altomonte, Amine Kamen, Udo Reichl, Yvonne Genzel

**Affiliations:** 1 Bioprocess Engineering, Max Planck Institute for Dynamics of Complex Technical Systems, Sandtorstr. 1, 39106 Magdeburg, Germany; 2 Department of Chemical Engineering, Polytechnique Montréal, Montréal, Québec Canada; 3 Repligen, Waltham, MA USA; 4 Department of Internal Medicine II, Klinikum rechts der Isar, Technische Universität München, Munich, Germany; 5 Department of Bioengineering, McGill University, Montréal, Québec Canada; 6 Chair for Bioprocess Engineering, Otto von Guericke University Magdeburg, Universitätsplatz 2, 39106 Magdeburg, Germany

**Keywords:** Tangential flow depth filtration, Alternating tangential flow filtration, Bioreactor, Perfusion, Recombinant VSV-based vectors, Oncolytics, Vaccines

## Abstract

**Abstract:**

Cell culture-based production of vector-based vaccines and virotherapeutics is of increasing interest. The vectors used not only retain their ability to infect cells but also induce robust immune responses. Using two recombinant vesicular stomatitis virus (rVSV)-based constructs, we performed a proof-of-concept study regarding an integrated closed single-use perfusion system that allows continuous virus harvesting and clarification.

Using suspension BHK-21 cells and a fusogenic oncolytic hybrid of vesicular stomatitis virus and Newcastle disease virus (rVSV-NDV), a modified alternating tangential flow device (mATF) or tangential flow depth filtration (TFDF) systems were used for cell retention. As the hollow fibers of the former are characterized by a large internal lumen (0.75 mm; pore size 0.65 μm), membrane blocking by the multi-nucleated syncytia formed during infection could be prevented. However, virus particles were completely retained. In contrast, the TFDF filter unit (lumen 3.15 mm, pore size 2–5 μm) allowed not only to achieve high viable cell concentrations (VCC, 16.4–20.6×10^6^ cells/mL) but also continuous vector harvesting and clarification. Compared to an optimized batch process, 11-fold higher infectious virus titers were obtained in the clarified permeate (maximum 7.5×10^9^ TCID_50_/mL).

Using HEK293-SF cells and a rVSV vector expressing a green fluorescent protein, perfusion cultivations resulted in a maximum VCC of 11.3×10^6^ cells/mL and infectious virus titers up to 7.1×10^10^ TCID_50_/mL in the permeate. Not only continuous harvesting but also clarification was possible. Although the cell-specific virus yield decreased relative to a batch process established as a control, an increased space-time yield was obtained.

**Key points:**

• *Viral vector production using a TFDF perfusion system resulted in a 460% increase in space-time yield*

• *Use of a TFDF system allowed continuous virus harvesting and clarification*

• *TFDF perfusion system has great potential towards the establishment of an intensified vector production*

**Supplementary Information:**

The online version contains supplementary material available at 10.1007/s00253-024-13078-6.

## Introduction

In the relentless pursuit to combat infectious diseases, recombinant vector-based vaccines produced in cell culture have gained popularity during the last decade. Compared to other vaccine platforms, viral vector-based vaccines retain their ability to infect cells, thereby inducing robust immune responses by increasing both humoral and cellular immunity (Ura et al. 2014). Successful large-scale applications of adenoviral-based vectors against SARS-CoV-2 (Mendonça et al. 2021) combined with their potential application against a wide array of infectious diseases, resulted in tremendous research efforts to develop new recombinant vector-based vaccines (accounting for 14% of global R&D vaccine landscape in 2023 (Yue et al. 2023)) and to improve current manufacturing processes. One such vector is based on the recombinant vesicular stomatitis virus (rVSV). Due to its broad tropism, fast replication kinetics to high titers, low viral pathogenicity, rare pre-existing anti-vector immunity in humans, and ease of genetic manipulation, rVSV has gained popularity for both vaccine and oncolytic applications (Ura et al. 2021). Replacing the native glycoprotein of rVSV to any glycoprotein of interest allows delivery of foreign antigens to elicit robust humoral and cellular immunity for vaccine applications, while simultaneously reducing the manufacturing-associated biosafety standards (e.g., for highly pathogenic viruses) (Zhang and Nagalo 2022). Various vaccine candidates manufactured using a rVSV production platform have shown prophylactic effects against Ebola (Suder et al. 2018), SARS-CoV-2 (Ura et al. 2021), Marburg virus (Jones et al. 2005), Lassa virus (Geisbert et al. 2005), Andes virus (Brown et al. 2011), hepatitis B virus (Cobleigh et al. 2010), *Yersinia pestis* (Palin et al. 2007), respiratory syncytical virus (Kahn et al. 2001), dengue virus (Lauretti et al. 2016), chikungunya virus (van den Pol et al. 2017), Nipah virus (DeBuysscher et al. 2016), Zika virus (Emanuel et al. 2018), human papillomavirus (Liao et al. 2008), and influenza virus (Roberts et al. 1998) in animal models; and some are being tested in clinical trials. Despite the well-demonstrated prophylactic efficacy, currently only one VSV-based vaccine against Zaire ebolavirus (rVSV-ZEBOV) gained FDA and EMA approval in 2019 (EMA 2019; FDA 2019). Usage of rVSV as oncolytic agents is particularly interesting, as its high cytopathogenicity, fast replication cycle, non-integration into the host genome, IFN-sensitivity, selective infection, and potent induction of apoptosis in cancer cells fulfill all critical features for virotherapy (Zhang and Nagalo 2022). Further genetic modification approaches such as pseudotyping innate glycoproteins with heterologous fusion glycoproteins, generating chimeric constructs that can convey fusogenic-based viral propagation, further enhance the oncolytic abilities of rVSV-based constructs (Abdullahi et al. 2018). One such novel rVSV-based construct contains the fusogenic mutant proteins of Newcastle disease virus (NDV) and has shown promising pre-clinical efficacy in various cancer models (Abdullahi et al. 2018; Krabbe et al. 2021).

To address the unprecedented demand, as well as the high input doses required for many rVSV-based therapies, current batch-based manufacturing strategies need to be intensified. Usage of suspension cell lines in chemically defined media allows for the design of processes that are easier to scale-up, for higher cell concentrations and for smaller footprints compared to traditional adherent-based manufacturing (Pelz et al. 2022). By the establishment of perfusion cultures, where depleted medium is continuously exchanged with fresh medium, while the cells are retained in the bioreactor by the use of a cell retention device, even higher cell concentrations can be achieved. Process intensification in perfusion mode to increase virus titers, cell-specific virus yields (CSVYs), space-time yield (STY), and volumetric virus productivity (VVP) have already been elaborated for different viruses and vectors such as for Zika virus (Nikolay et al. 2018), influenza A virus (IAV) (Wu et al. 2021), lentiviral vectors (LV) (Tran and Kamen 2022; Tona et al. 2023), adeno-associated virus (AAV) (Mendes et al. 2022), rVSV-COV-2 (Yang et al. 2023), rVSV-NDV (Göbel et al. 2023a), and modified vaccinia virus Ankara (Gränicher et al. 2021a; Gränicher et al. 2021b). However, by targeting higher cell concentrations, the CSVY might be decreased (high-cell density effect) thus lowering the STY and VVP (Bock et al. 2011; Nadeau and Kamen 2003).

For high-cell concentration cultivations, membrane-based systems, i.e., alternating tangential flow (ATF) and tangential flow filtration (TFF) modules are widely employed. One major drawback of these systems is the risk of filter fouling (despite the self-cleaning backflush of the ATF) and retention of virus particles, which leads to unwanted accumulation of virus inside the bioreactor within the cell environment until full harvest of the bioreactor broth is possible (Genzel et al. 2014; Hadpe et al. 2017; Nikolay et al. 2020; Nikolay et al. 2018; Tona et al. 2023; Vázquez-Ramírez et al. 2019). As virus release typically leads to cell lysis, cell debris and DNA increase over time, equally increasing viscosity of the culture broth and further increasing the risk of membrane clogging. Prolonged retention inside the bioreactor can have a negative impact on virus infectivity due to a release of cellular proteases, adsorption of virions to cellular debris, and viral temperature sensitivity (Aunins 2003; Eccles 2021; Genzel et al. 2010; Göbel et al. 2023a; Gränicher et al. 2020; Wu et al. 2021). Additionally, fusogenic oncolytic constructs such as rVSV-NDV that lead to the formation of large multi-nucleated syncytia (>100 μm) in perfusion cultures are likely to block the small lumen sizes of commonly used hollow-fiber membranes (Göbel et al. 2023a). One approach to overcome this issue is the use of non-membrane-based systems such as an acoustic filter (Göbel et al. 2023a; Gränicher et al. 2020; Manceur et al. 2017) or an inclined settler (Coronel et al. 2020); however, these systems are often complex or not compatible with industrial size bioreactors and have a lower cell retention efficiency. Alternatively, membranes that allow virus particles to pass through could be used. This has been recently demonstrated for the production of IAV defective interfering particles utilizing a tubular membrane (VHU, pore size ~10 μm) coupled to an ATF system (Hein et al. 2021). Using tangential flow depth filtration (TFDF) perfusion modules (pore size 2–5 μm), continuous harvest of LV as well as AAV has been already shown (Mendes et al. 2022; Tona et al. 2023; Tran and Kamen 2022). This allows for shorter residence times of infectious virus particles and the possibility to immediately store harvested material in cooled tanks to increase virus stability and, thus, virus yields and productivity. For instance, continuous virus harvest using an acoustic settler for IAV production enhanced CSVY and VVP by a factor of 1.5 relative to a perfusion run with a hollow fiber membrane with PES (0.2 μm cut-off) (Gränicher et al. 2020). Moreover, the TFDF module can combine continuous virus harvest and clarification in a single step, reducing the number of unit operations and therefore saving time and money (Mendes et al. 2022). All in all, this could allow for a direct integration of upstream (virus production) and downstream processing (virus purification), further reducing costs while increasing flexibility and productivity (Gränicher et al. 2021a; Moleirinho et al. 2020).

In this study, we evaluated the applicability of the TFDF perfusion system as a novel cell retention device and virus transmission away for the host cell environment for both perfusion cultivation and continuous harvest filtration with clarification (turbidity reduction) in a single operation. The process intensification of the production of two different rVSV-based vectors, one which induces classical cytopathic effects and one that mediates cell fusion reactions, was compared to optimized batch processes.

## Materials and methods

### Cell lines, media, and viral seed stock

Baby hamster kidney (BHK-21) cells (CEVA Animal Health) were cultivated in protein expression medium (PEM) (Gibco, USA) supplemented with 4-mM pyruvate and 8 mM L-glutamine (Sigma-Aldrich, USA). Cells were sub-cultured to 0.5 × 10^6^ cells/mL twice a week using vented, baffled 125-mL shake flasks (50 mL working volume (WV)) at 37 °C and 5% CO_2_ with controlled agitation (185 rpm, shaken diameter of 50 mm, Infors HT, Switzerland). Human embryonic kidney (HEK293-SF) cells were kindly provided by National Research Council of Canada (NRC, Montreal, Canada) and grown in vented, non-baffled 125-mL shake flasks (TriForest Enterprises, USA) in HyClone HyCell TransFx-H medium (Cytiva, USA) supplemented with 6 mM glutamine and 0.1% Koliphor P188 (Merck, USA). HEK293-SF cells were passaged three times a week at 0.2×10^6^ or 0.5×10^6^ cells/mL and grew in a humified Multitron orbital shaker (shaking diameter of 25 mm, Infors HT, Switzerland) at 135 rpm, 37 °C, and 5% CO_2_. Adherent Huh7 cells were cultivated at 37 °C and 5% CO_2_ in T75 flasks in high glucose Dulbecco’s Modified Eagle Medium (DMEM, Gibco, USA), supplemented with 1 mM sodium pyruvate (Gibco, USA), 1× non-essential amino acids (Gibco, USA), and 10% FCS. Adherent HEK293 cells were maintained in T75 or T175 flasks in DMEM (Wisent Bioproducts, Canada) supplemented with 10% FCS (Wisent Bioproducts, Canada) at 37 °C and 5% CO_2_ in a humidified incubator and sub-cultured twice a week by using TrypLE Express (Thermo Fisher Scientific, USA). Adherent AGE1.CR.pIX cells were maintained at 37 °C, 5% CO_2_ in T75 flasks in DMEM-F12 medium (Gibco, USA).

For infections, the previously described BHK-21-derived virus seed (rVSV-NDV) with a titer of 1.33×10^9^ 50% tissue culture infectious dose (TCID_50_)/mL was used (Göbel et al. 2023a). Moreover, we used rVSV-green fluorescent protein (GFP) virus seed with a titer of 2.12×10^9^ TCID_50_/mL, which was derived from suspension HEK293 cells and kindly provided by NRC. Aliquots of the stocks and clarified samples were stored at −80 °C and were used once for each experiment or assay to prevent loss of infectivity due to repeated freeze-thaw cycles.

### Perfusion mode production of rVSV-NDV in an orbitally shaken bioreactor

To evaluate applicability of membrane-based cell retention for production of fusogenic oncolytic viruses, a perfusion run employing a modified alternating tangential flow filtration device (mATF) as a cell retention device was carried out. Here, a SB10-X orbital shaken bioreactor (OSB) (Adolf Kühner AG) was used with the novel 3 L modular adapter and standard 3-L single-use bags. BHK-21 cells were inoculated at 0.9×10^6^ cells/mL with 2.4 L WV at 37 °C and shaken at 100 rpm (50-mm shaking diameter). Aeration was solely carried out through headspace gassing using 300 mL/min air/O_2_. By automatic adjustments of the gas composition in the output flow, dissolved oxygen (DO) and pH were controlled at 80% and 7.20, respectively. Perfusion was initiated once viable cell concentration (VCC) reached 4−10^6^ cells/mL. The perfusion rate was manually increased over time to maintain a cell-specific perfusion rate (CSPR) of 115 pL/cell/day. For virus production, temperature was decreased to 34 °C, and the perfusion rate was set to 1.8 RV/day. Coupling of the 3-L single-use bag to a hollow fiber membrane (0.65-μm modified polyethersulfone (mPES), 1075 cm^2^, Repligen, USA) connected to the mATF (Repligen, USA) was carried out as described previously (Coronel et al. 2019). Exchange flow rates of the diaphragm pump were set to 1.5 L/min. As the ATF2 module was placed below the SB10-X, a height differential of 40 cm was set, while other parameters were kept as given by the supplier.

### Perfusion mode production of rVSV-NDV in a STR

**Fig. 1:**
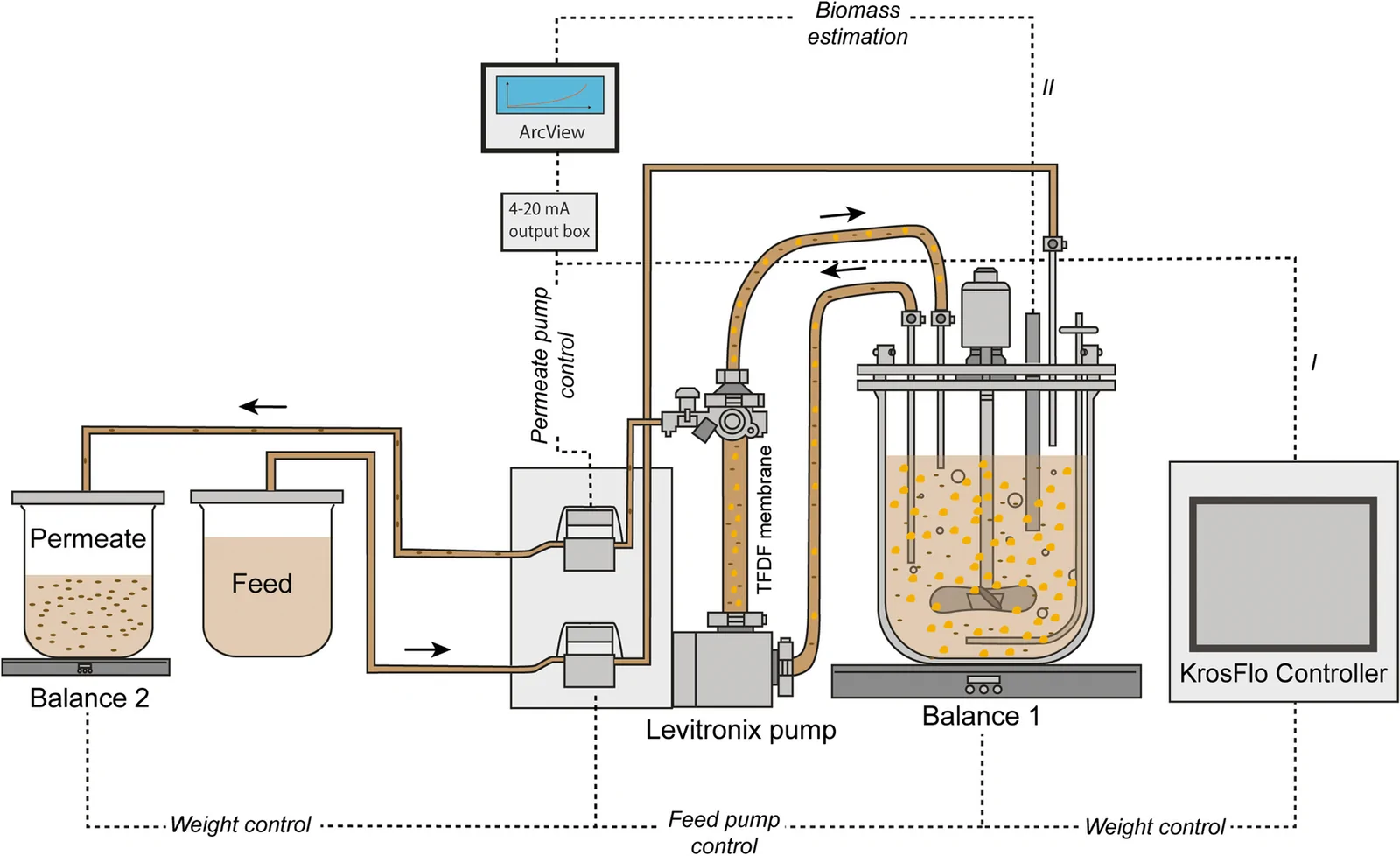
Scheme of the TFDF setup for perfusion cultivations with manual adjustment (I) or automated (II) perfusion control (adapted from (Göbel et al. 2022b)). BHK-21 cells were continuously pumped through a 30-cm^2^ TFDF cartridge (pore size 2–5.0 μm) by a levitronix impeller pump in unidirectional flow. Flow rates for feed and inlet were controlled using balances and KrosFlo’s integrated flow sensor, respectively. For the cell growth phase, the permeate flow was either manually adjusted (I, using the KrosFlo controller) or controlled (II, using a capacitance probe). Larger orange circles indicate cells, black ellipses indicate virus particles, and dashed lines indicate different types of signal transmission

Bioreactor perfusion cultivations were performed using a 3-L stirred tank bioreactor (STR) (DASGIP, Eppendorf AG, Germany) equipped with two pitched blade impellers (50-mm diameter; 180 rpm) and a L-drilled hole sparger, as well as a microsparger for gas supply. The DO setpoint of 50% was maintained by varying the gas flow rates (3–9 L/h) and the percentage of O_2_ in the gas mixture (21–100%). pH was controlled at 7.20 by sparging CO_2_. Temperature was set to 37 °C for the growth phase and 34 °C for the infection phase. BHK-21 cells were inoculated at 0.8×10^6^ cells/mL at 1.3 L WV, and the cells were grown in batch mode until a VCC of 4.0×10^6^ cells/mL was reached. Then, perfusion was started, and medium was exchanged with a CSPR of 130 pL/cell/day for BHK-21 cells. For cell retention, a 30-cm^2^ TFDF cartridge (polypropylene and polyethylene terephthalate, pore size 2–5 μm, Repligen) connected to a Krosflo TFDF system (Repligen) was used. Using the KrosFlo’s integrated flow sensor and weight control system, the recirculation rate and WV were maintained at 0.9 L/min and 1.3 L, respectively. Permeate flow rates were either updated daily based on VCC and the CSPR or controlled automatically through a capacitance probe and pre-amplifier connected to the ArcView controller 265 (Incyte Hamilton, USA), as described previously (Göbel et al. 2023a). A “cell factor” of 0.25 was used to set the cell volume-specific perfusion rate (CVSPR) of 0.06 pL/μm^3^/day. At time of infection (TOI), an RV was exchanged by temporarily increasing the permeate flow rate to 8–10 mL/min, and cells were subsequently infected with a multiplicity of infection (MOI) of 1E-4. After infection, permeate flow was paused for up to 2 h and then fixed to 1.8 RV/day (1.7 mL/min). The clarified permeate was collected continuously into sterile polyethylene terephthalate bottles, previously filled with sucrose equal to 5% final concentration, at room temperature. The final one-step bioreactor harvest was carried out using a modified concentration-diafiltration-concentration (C1/DF/C2) process (Table S1), where the permeate flow was paused during the diafiltration step. Furthermore, either sterile PBS (TFDF1) or supplemented medium (TFDF2) was used for diafiltration. The overall setup including devices for control of perfusion is shown in Fig. 1.

### Other analytics for rVSV-NDV experiments

VCC and viability were quantified using the automated cell counter ViCell (Coulter Beckman, USA). A pH7110 potentiometer (Inolab, USA) was used to measure off-line pH, and metabolite concentrations (glucose, lactate, glutamine, glutamate, ammonium) were determined by a Cedex Bio Analyzer (Roche, Switzerland). Titration of rVSV-NDV was carried out using adherent AGE1.CR.pIX cells and the previously described TCID_50_ assay (Göbel et al. 2022a, 2022b). Oncolytic viral potency was confirmed using the previously described half maximal inhibitory concentration (IC50) potency assay in Huh7 cells (Göbel et al. 2023a; Göbel et al. 2022a). Taking into account only the error of the TCID_50_ assay (−50%/+100% on a linear scale), the CSVY was calculated as previously described by Gränicher et al. 2020. Total DNA and protein were quantified using the Quant-iT™ PicoGreen dsDNA assay kit (Thermo Fisher Scientific, USA) and with Pierce™ BCA assay kit (Thermo Fisher Scientific, USA), respectively, according to the manufacturer’s instructions. The solution turbidity of bioreactor and permeate samples was measured using a turbidimeter (2100 Qis Portable, HACH).

### Production of rVSV-GFP in shake flasks

For production of rVSV-GFP in batch mode using shake flasks, HEK293-SF cells growing in exponential growth phase were centrifuged (300×*g*, 5 min, room temperature (RT)), supernatant was discarded, and cells were resuspended in penicillin/streptomycin (Pen/Strep)-containing fresh medium. Subsequently, cells were seeded at 0.4×10^6^ cells/mL into a shake flask and grown to 1.1×10^6^ cells/mL. Cells were infected at an MOI of 1E-3, and temperature was reduced from 37 to 34 °C. Supernatant samples were centrifuged (1200×*g*, 5 min, 4 °C) and stored at −80 °C for analytics.

### Batch mode production of rVSV-GFP in a STR

Production of rVSV-GFP in batch mode was conducted in a 3 L (2100 mL WV) or 1 L (700 mL WV) STR (Applikon Biotechnology, Netherlands), which was equipped with two (3 L STR) or one (1 L STR) marine impeller(s) (100 rpm). Continuous surface aeration (12.5 mL/min), with sparging of O_2_ (microsparger) when necessary, was used to control dissolved oxygen above 40% DO. The pH set point of 7.15 was controlled by CO_2_ injection into the headspace of the bioreactor or addition of 9% NaHCO_3_. HEK293-SF cells cultured in shake flasks were centrifuged (300×*g*, 5 min, RT), resuspended in fresh Pen/Strep-containing medium and inoculated at about 0.3×10^6^ cells/mL into the STR. HEK293-SF cells were grown at 37 °C up to 1.2–1.3×10^6^ cells/mL. Prior to infection, the temperature was reduced to 34 °C, and cells were infected at an MOI of 1E-3.

### Perfusion mode production of rVSV-GFP in a STR

For perfusion mode production of rVSV-GFP, a 3 L STR was used at 2100 mL WV. Standard bioreactor setup and process parameters are similar to those described in the previous chapter. Before inoculation, recirculation was started with a recirculation rate of 1.0 L/min. HEK293-SF cells growing in shake flasks in the exponential growth phase were centrifuged (300×*g*, 5 min, RT), resuspended in fresh Pen/Strep-containing medium, and seeded at 0.8×10^6^ cells/mL. After a cell growth phase in batch mode for 24 h, perfusion was initiated with a permeate flow rate of 0.9 mL/min (0.6 RV/d). In the following, the permeate flow rate was adjusted manually based on a CSPR of 115 pL/cell/day. The bioreactor weight was used to control the feed flow rate. Prior to infection, one RV with medium was exchanged by temporarily increasing the permeate flow rate to 20–33 mL/min. Temperature was reduced to 34 °C, and HEK293-SF cells (10×10^6^ cells/mL) were infected at an MOI of 1E-3. Following infection, the permeate flow was stopped for 1 h, and then set at a constant rate of 2.2 mL/min (1.5 RV/day). Supernatant samples were taken from the bioreactor and permeate line. After infection, the accumulated clarified permeate in the permeate bottle was stored on ice and transferred to a storage bottle (stored at 4 °C) at each sample taking. Lastly, a final “pool sample” was taken from the storage bottle containing the entire volume of collected permeate, without the final harvest step. For final one-step harvest at 31-h post infection (hpi), the recirculation rate was increased to 2.1 L/min to prevent filter fouling and promote self-cleaning. In more detail, a C1/DF/C2 process was conducted (Table S2). In the first step, 980 mL cell broth was pumped (31.3 mL/min) from the bioreactor through the TFDF filter into the harvest bottle. Next, 1049 mL medium was pumped (31.3 mL/min) into the bioreactor for washing, while harvest continued. In the last step, feeding of medium was stopped, while another 491 mL of suspension was pumped from the bioreactor through the TFDF filter into the harvest bottle. Supernatant samples were centrifuged (1200*×g*, 5 min, 4 °C) and stored at −80 °C until further analysis. For metabolite measurements, virus was removed using a Vivaspin 500 (Cytiva, USA, molecular weight cut-off of 100 kDa, 10,000×rpm, 5 min).

### Other analytics for rVSV-GFP experiments

Quantification of VCC, viability, and cell diameter was performed using a cell counter (Vi-Cell^TM^ XR, Beckman Coulter, USA). Metabolite concentrations were quantified by Bioprofile^®^ FLEX 2 (Nova Biomedical, USA). A TCID_50_ assay was used to quantify infectious virus titer of rVSV-GFP samples. In brief, adherent HEK293 cells were seeded at a concentration of 0.2×10^5^ cells/well (100 μL per well) in 96-well plates using DMEM (10% FCS, 1% Pen/Strep), followed by incubation for 24 h at 37 °C. Prior to infection, medium was gently removed from wells by a vacuum aspirator (8-channel adaptor). Cells were infected (infection medium: 2% FCS, 1% Pen/Strep) with eight replicates (100 μL/well) per dilution (serial dilution of 1:10) and incubated for 7 days at 34 °C. Wells containing cytopathic effect under standard light microscope were considered as positive. Spearman/Kärber method was used to calculate TCID_50_ titer. Each sample was quantified twice in independent TCID_50_ assays.

### Calculations

Metabolite consumption/production rates (*q*_*s*_), CSVY (TCID_50_/cell), volumetric virus productivity (VVP; TCID_50_/L/day), space-time yield (STY, TCID_50_/L/day), and cell retention efficiency (CRE) were calculated as follows:

1 $${\textrm{q}}_{\textrm{s}}=\frac{{\mu}}{{{Y}}_{{x}/{s}}}$$

2 $${Y}_{x/s}=\frac{\textrm{x}\left({t}_n\right)-\textrm{x}\left({t}_{n-1}\right)}{c_s\left({t}_{n-1}\right)-{c}_s\left({t}_n\right)}$$

where *μ* is the cell-specific growth rate (1/h), *Y*_*x/s*_ is the biomass yield, *x* is the VCC (cells/mL) at the cultivation time *n* (*t*_*n*_), and *c*_*s*_ the metabolite concentration (mM).

*vir*_acc_ is the accumulated number of infectious virus particles (TCID_50_) and was calculated for different production modes as follows:

3 $$\textrm{Batch}:{vir}_{\textrm{acc}}={C}_{vir, BR}\times {V}_{BR}$$

*C*_*vir*, *BR*_ is the infectious virus concentration (TCID_50_/mL) in the bioreactor. *V*_BR_ (mL) is the working volume of the cultivation vessel.

4 $$\textrm{ATF},\textrm{AS}:{\textrm{vir}}_{\textrm{acc}}={C}_{vir, BR}\times {V}_{BR}+\sum \frac{\left({C}_{vir,P,n}+{C}_{vir,P,n-1}\right)}{2}\times {V}_H$$

*C*_*vir*, *P*, *n*_ and *C*_*vir*, *P*, *n* − 1_ are the infectious virus concentrations in the permeate between *t*_*n*_ and *t*_*n*−1_, respectively. *V*_*H*_ represents the harvest volume collected between *t*_*n*_ and *t*_*n*−1_.

5 $$\textrm{TFDF}:{\textrm{vir}}_{\textrm{acc}}={C}_{vir, FH}\times {V}_{FH}+\sum {C}_{vir,B,n}\times {V}_{B,n}$$

*C*_*vir*, *FH*_ is the infectious virus concentrations of the final harvest step. *V*_*FH*_ represents the volume of the final harvest step. *C*_*vir*, *B*, *n*_ and *C*_*vir*, *B*, *n* − 1_ are the infectious virus concentrations in the harvest bottles, which were exchanged every sample time point for rVSV-NDV. *V*_*B,n*_ represents the volume in the harvest bottle. For the TFDF run with rVSV-GFP, the collected permeate (stored at 4 °C) was sampled combined, and final harvest step was sampled individually. For the TFDF run with rVSV-NDV, all harvest bottles were sampled individually and not combined.

6 $$\mathrm{CSVY}=\frac{{\textrm{vir}}_{\textrm{acc}}}{{{x}}_{{max}}\times {{V}}_{{BR}}}$$

7 $$\textrm{VVP}=\frac{{\textrm{vir}}_{\textrm{acc}}}{{{V}}_{{acc}}\times {{t}}_{{t}{ot}}}$$

8 $$\textrm{STY}=\frac{{\textrm{vir}}_{\textrm{acc}}}{{{V}}_{{BR}}\times {{t}}_{{t}{ot}}}$$

*x*_*max*_ (cells/mL) is the maximum viable cell concentration reached post-infection in the cultivation vessel. *V*_*acc*_ (mL) is the accumulated medium spent during the entire process, and *t*_*tot*_ (h) is the total process time until maximum *vir*_*tot*_ is reached.

Percentage of infectious virus (*P*_*Perm*_) passing through the membrane was calculated as follows:

9 $${P}_{Perm}=\frac{1}{n}\sum \left(\frac{C_{vir,P,n}}{C_{vir, BR,n}}\right)\times 100\%$$

Cell retention efficiency (CRE), shear rate (γ), and permeate flux (J) were calculated as follows:

10 $$\textrm{CRE}=\left(\textrm{1}-\frac{{{x}}_{{H}}}{{{x}}_{{Br}}}\right)\times \textrm{100}$$

11 $$\gamma =\frac{4\times Q}{z\times \pi \times {R}^3}$$

12 $$J=\frac{\dot{V_p}}{A}$$

where *x*_*H*_ and *x*_*BR*_ are the measured VCC in the permeate line and in the bioreactor, respectively. *Q* represents the volumetric recirculation rate (m^3^/s, based on the exchange flow rate of the ATF system or recirculation rates of the TFDF system), *R* the internal radius of the fiber (m), and *z* the number of hollow-fibers of the ATF/TFDF membrane. The permeate flux is calculated as the ratio of the permeate flow rate $\dot{V_p}$(L/h) to the total filtration area of the hollow-fiber membrane *A* (m^2^).

## Results

### rVSV-NDV production using mATF

**Fig. 2:**
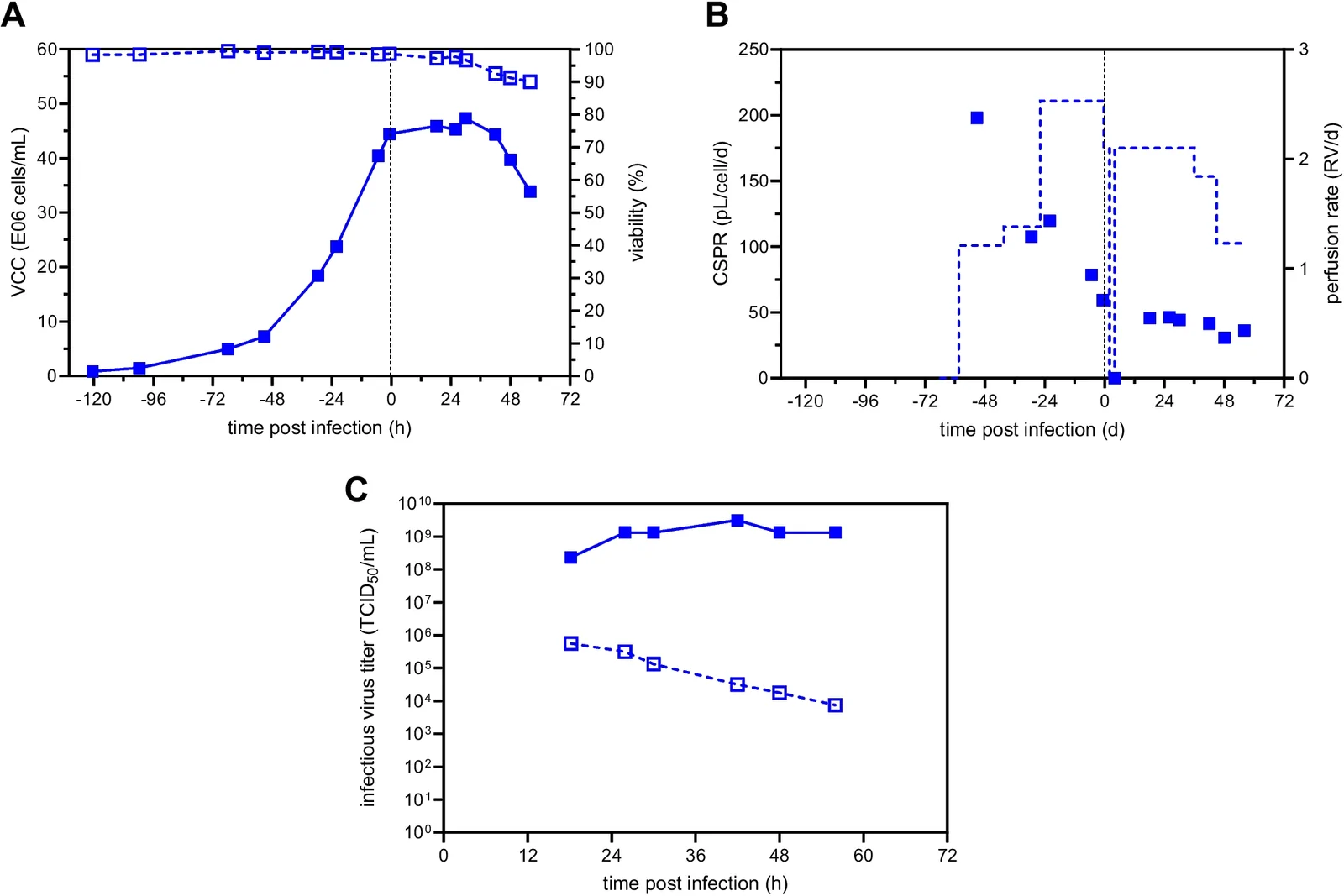
rVSV-NDV production in BHK-21 cells in perfusion mode using a SB10-X equipped with a 3-L modular adapter and connected to a mATF. BHK-21 cells were inoculated at 0.9×10^6^ cells/mL, and perfusion was started 56 h after batch growth phase. The perfusion rate was adjusted manually over time. For cell retention, a 0.65-μm mPES hollow fiber membrane was used. Infection was carried out once a VCC of 44.5×10^6^ cells/mL was reached (MOI of 1E-4), temperature was reduced to 34 °C, and perfusion was paused for 4 h. **A** VCC (full symbols) and viability (hollow symbols). **B** Cell-specific perfusion rate (full symbols) and perfusion rate (dashed lined, weight of collected permeate divided by WV). **C** Infectious virus titer measured inside the bioreactor (full symbols) and the permeate line (hollow symbols)

To evaluate the virus retention of commonly used hollow-fiber membranes and the applicability for fusogenic oncolytic viruses forming large multi-nucleated syncytia, an initial perfusion run using the novel 3-L modular adapter on a SBX-10 OSB with a 0.65-μm mPES membrane connected to a mATF was carried out. Previously, it has been shown that OSB and STR bioreactors perform similarly for cell growth and, therefore, to demonstrate all possible applications, an OSB was chosen (Coronel et al. 2019; Göbel et al. 2023b) for this study. To prevent potential syncytia from blocking the hollow fibers, a membrane with fiber lumen larger than previously observed syncytia (120–140 μm) was chosen (0.75-mm internal fiber lumen). Following inoculation at 0.8×10^6^ cells/mL at 2.4 L WV, perfusion was initiated once VCC reached 4×10^6^ cells/mL. Manual adjustment of the perfusion rate allowed growth to 44.5×10^6^ cells/mL with viabilities above 98% (Fig. 2A) without any limitations in glucose and glutamine (data not shown); however, the CSPR could not be controlled stably (Figure 2B). In order to conserve medium, no medium exchange prior to infection was carried out for this run. Cells were directly infected at an MOI of 1E-4 once cells reached a VCC of 44.5×10^6^ cells/mL, temperature was reduced to 34 °C, and perfusion was paused for 4 h. Following re-initiation of perfusion, VCC stagnated until 36 hpi, after which viability and VCC slowly declined (Fig. 2A). Maximum infectious virus titers of 3.2×10^9^ TCID_50_/mL were reached at 42 hpi in the bioreactor, corresponding to a CSVY of 67 TCID_50_/cell and a VVP of 4.0×10^10^ TCID_50_/L/day (Table 1).

**Table 1:** Comparison of rVSV-NDV production in BHK-21 cells for different production modes. Cell growth parameters were determined before infection.

	Batch_a_	Perfusion AS_a_	ATF	Perfusion TFDF	
				TFDF1	TFDF2
Bioreactor vessel	STR (1 L)	STR (1 L)	OSB (3 L)	STR (3 L)	STR (3 L)
Cell-specific growth rate (1/h)	0.033	0.019±0.003	0.031	0.027	0.030
Doubling time (h)	21.0	36.1±6.5	22.1	25.9	23.0
qGlc (10^−11^ ×mmol/(cell/h))	5.1±0.9	6.5±2.1	8.25	8.06	15.2
qGln (10^−11^ ×mmol/(cell/h))	3.0±0.7	3.1±2.0	2.42	2.21	3.21
Max. VCC p.i. (10^6^ cells/mL)	3.2±0.3	29.7±2.4	44.5	20.6	16.4
Max. infectious virus titer (10^8^ TCID_50_/mL)	5.0±0.9	15.8±11.7	31.6	75.0	56.2
CSVY (TCID_50_/cell)	161±40	118±11	67	365	342
VVP (10^10^ TCID_50_/L/d)	9.4±2.6	3.9±0.6	4.0	8.8	11.5
STY (10^10^ TCID_50_/d)	13.4±3.7	29.0±5.2	43.0	75.5	95.4
Used medium (L)	0.7	6.9±1.9	25.1	11.0	9.3
dsDNA level at optimal harvest time point (μg/mL)	n.d.	14.0±0.5	n.d.	7.5	13.9
Protein level at optimal harvest time point (mg/ml)	n.d.	0.5	n.d.	2.0	2.5

*qGlc*, cell-specific glucose consumption rate; *qGln*, cell-specific glutamine consumption rate; *max*., maximum; *VCC*, viable cell concentration; *p.i*., post infection; *n.d*., not determined. Optimal harvest time point was defined as time point when the maximum infectious virus titer was reached in the supernatant. *a*: Values taken from Göbel et al. 2023a, 2023b carried out as biological replicates with *n*=2

To assess the virus retention by the 0.65-μm hollow fiber membrane, samples were taken from the bioreactor and the permeate line, respectively. Already at 18 hpi, infectious virus titers in the permeate were significantly reduced compared to the bioreactor (>2 log), corresponding to 97.0% retention. With increasing process time, virus retention further increased to up to 5 log or nearly 100% (Fig. 2C). Surprisingly, the high flow rate through the hollow fiber membrane of 1.5 L/min and the resulting high shear stress of 5490 1/s neither impacted cell growth (Fig. 2A) nor virus replication (Fig. 2C) and did not prevent the formation of large multi-nucleated syncytia (50–75 μm, Figure S4).

### rVSV-NDV production using TFDF

**Fig. 3:**
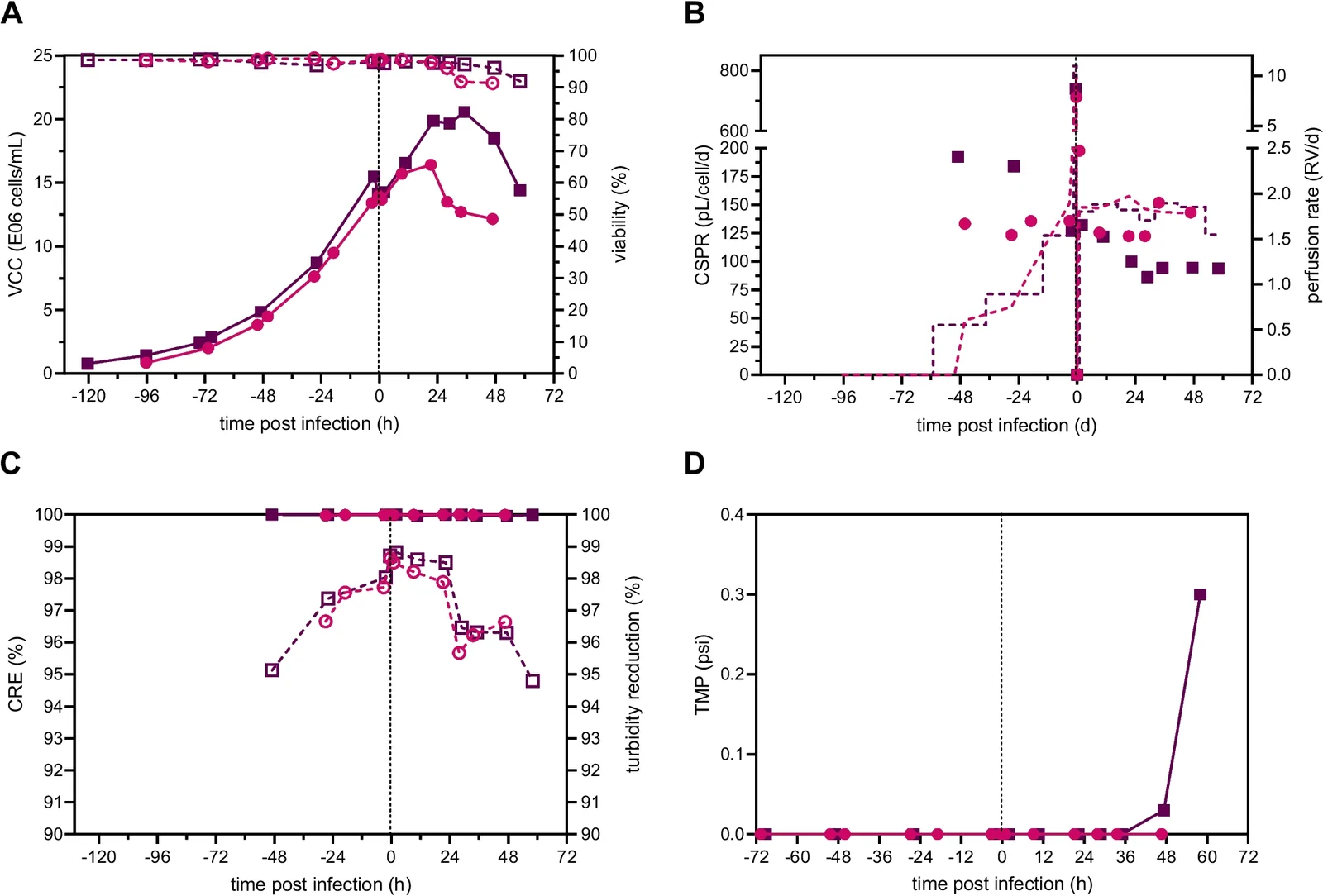
Critical process parameters of rVSV-NDV production in BHK-21 cells in perfusion mode using a 3-L STR coupled to a TFDF system. BHK-21 cells were inoculated at 0.8×10^6^ cells/mL, and perfusion was started 46–51 h after batch growth phase by utilizing a TFDF cartridge (pore size 2–5 μm). The first TFDF run (TFDF1, purple squares) was adjusted manually, while the second TFDF run (TFDF2, pink circles) was controlled based on the biovolume measured by a capacitance probe. Infection was carried out once a VCC of 14×10^6^ cells/mL was reached (MOI of 1E-4), temperature was reduced to 34 °C, and perfusion was paused for 1–2 h. **A** Viable cell concentration (full) and viability (empty). **B** Cell-specific perfusion rate (full) and perfusion rate (dashed lined, weight of collected permeate divided by WV). **C** Cell retention efficiency (full) and turbidity reduction (empty) of TFDF membrane during growth and infection. **D** Transmembrane pressure (TMP) during cell growth and infection phase. The dashed line indicates the time of infection

In a next step, we carried out two rVSV-NDV production runs in a 3 L STR using TFDF as the cell retention device for perfusion as a proof of concept for continuous virus harvesting with clarification, targeting a VCC at TOI of 14×10^6^ cells/mL. For both TFDF runs, BHK-21 cells were inoculated at 0.8×10^6^ cells/mL in 1.3 L WV and grown in batch mode until a VCC of 4×10^6^ cells/mL was reached. In a next step, after initiation of perfusion, cell broth was recirculated with a constant recirculation rate of 0.9 L/min, corresponding to a shear rate of 1650 1/s. The perfusion rate was either manually adjusted (TFDF1) or controlled based on capacitance (TFDF2), where the signal was correlated to the biovolume. Both control strategies, enabled cells to grow up to 14×10^6^ cells/mL with viabilities above 97%, but with a slightly reduced cell-specific growth rate compared to the initial batch phase (*μ*=0.026 1/h to *μ*=0.035±0.005 1/h). A linear correlation of the permittivity signal with the VCC was obtained during the growth phase for both runs, enabling the monitoring of cell growth throughout the cultivation and the accurate control of the perfusion rate at the pre-defined CSPR of 130±5 pL/cell/day for TFDF2 (Figure S1, Fig. 3B). As the perfusion rate for TFDF1 was adjusted manually in a step-wise manner (Fig. 3B), the CSPR did not stay stable and varied around 168±36 pL/cell/day during cell growth (Fig. 3B). This resulted in a 15% lower total medium consumption for the growth phase for the capacitance-based control compared to the manual adjustment (Table 1). Interestingly, the glucose uptake rate of TFDF2 was 1.9-fold higher compared to TFDF1 (Table 1) resulting in an increase in lactate concentration up to 20 mM compared to 10 mM for TFDF1 (Figure S2B). However, all measured metabolite concentrations were still in the expected range with no notable limitations (Figure S2), and substrate uptake rates of TFDF 1 were comparable to both acoustic settler and ATF cultivations (Table 1, Göbel et al. 2023a, 2023b). For both runs, high cell retention rates of >99.9% and turbidity reductions >95% were achieved over the entire cultivation period (Fig. 3C). Transmembrane pressure (TMP) remained low (<0.1 psi) throughout the entire run, only showing a slight increase to 0.3 psi at 60 hpi for TFDF1 (Fig. 3D).

**Fig. 4:**
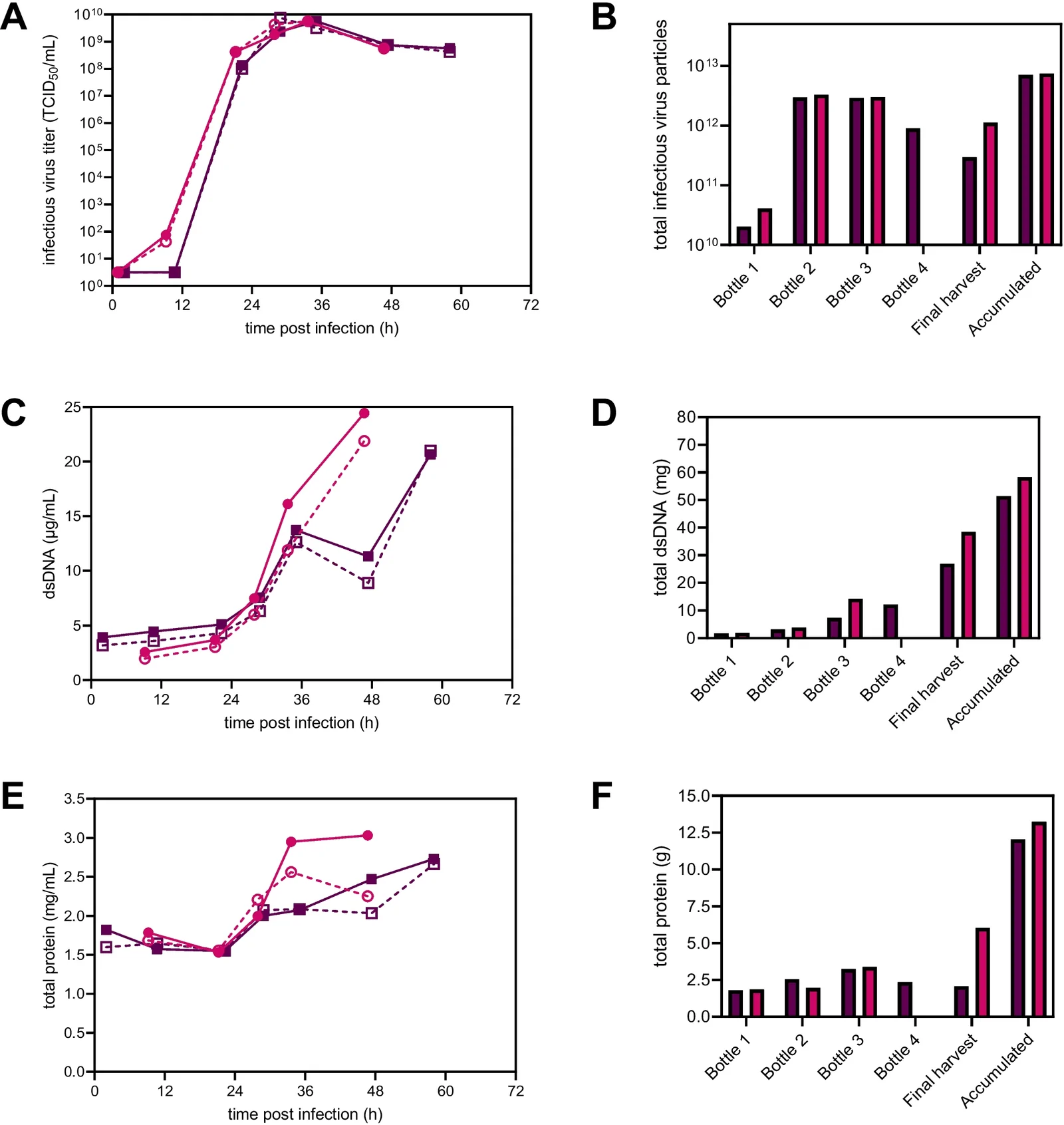
rVSV-NDV production in BHK-21 cells in perfusion mode for TFDF1 (purple squares) and TFDF2 (pink circles). Time course of **A** infectious virus titer (TCID_50_/mL), **C** double-stranded DNA (μg/mL), and **E** total protein (mg/mL) measured both in the reactor (full symbols) and permeate line (empty symbols). The TFDF membrane allowed a continuous harvesting of rVSV-NDV via the permeate. **B**, **D**, **F** The permeate was collected in multiple fractions (“Harvest bottle 1-4”), which were exchanged every 12–24 h to prevent loss of virus infectivity. Bottle 1 refers to permeate collected from 0 to 24 hpi, Bottle 2 24–36 hpi, Bottle 3 36–48 hpi, and bottle 4 48–60 hpi. “Final harvest” refers to material recovered in the final harvest step (concentration 1, diafiltration; final concentration, 2). “Accumulated” refers to the accumulated yields from all bottles and the final harvest

Prior to infection of cells with rVSV-NDV at an MOI of 1E-4, the medium was completely exchanged using a flow rate of 10 mL/min, and the temperature was reduced to 34 °C. Compared to the mATF perfusion, the permeate flow was only paused for 1–2 h (compared to 4 h) before setting the perfusion rate to a constant rate of 1.8 RV/day, as no loss of infectious virus particles into the permeate was observed. For both runs, cells continued to grow for 24–36 hpi up to a VCC of 20.5×10^6^ cells/mL and 16.4×10^6^ cells/mL displaying high viabilities above 96%. By sampling the bioreactor and permeate line, virus retention by the TFDF membrane can be assessed. For both runs, maximum titers of 7.5×10^9^ TCID_50_/mL and 5.6×10^9^ TCID_50_/mL were reached in the permeate at 29–34 hpi, respectively (Fig. 4A). Overall, infectious titers were very similar between the bioreactor and permeate line, indicating that rVSV-NDV was not retained by the TFDF membrane. On average, the percentage of infectious virus passing through the membrane was calculated as 124% and 118% for TFDF1 and TFDF2, respectively. Determination of the actual recovery was made by integration of the permeate line samples over the collected volume, considering the changing concentrations within the permeate line and collected volume (Figure S3). Here, the area under the curve represents the maximum available amount of infectious virus particles that can be recovered. Maximum theoretical values were compared to the sum of the actual empirical values for the collected fractions (“Accumulated” Fig. 4B). For both runs, recoveries of 78% were obtained, most likely due to the partial loss of functionality while storing at RT. As expected due to the large pore sizes, impurity concentrations were very similar between the bioreactor and permeate line (Fig. 4C and E) and gradually increased during the infection, reaching maximum values at the time of final harvest, when viabilities were reaching 90% (Fig. 4D and F).

Interestingly, monitoring the bioreactor samples by bright field microscopy showed limited syncytia formation, reaching sizes of 25–38 μm in diameter, for 29 hpi onwards for TFDF1. In contrast, more pronounced syncytia formation was found for TFDF2, where scattered clusters of fused cells appeared between 18 and 33 hpi, reaching sizes of 50–100 μm in diameter (Figure S4). For the final one-step harvest, a modified C1-DF-C2 process was utilized using the same TFDF membrane as for cell growth and infection. For TFDF1, filter fouling was observed for the harvest at 60 hpi, using sterile PBS for diafiltration, and the membrane was completely blocked during the second concentration step after removal of 1100 mL (Figure S5). By carrying out the harvest 12 h earlier at 48 hpi, further increasing the recirculation rate to promote self-cleaning of the membrane while simultaneously reducing the permeate flow, and using medium for diafiltration, filter fouling was prevented, and the TMP was kept below 2 psi for the majority of the harvest for TFDF2 (Figure S5).

Finally, the TFDF production was compared with the ATF perfusion run using the mATF and a previously described optimized batch process and perfusion process using an acoustic settler (AS) as the cell retention device (Table 1). All perfusion systems achieved a higher infectious virus concentration compared to an optimized batch process infected at 2×10^6^ cells/mL, with both TFDF runs being more than ten times higher compared to batch and more than 3.5 times higher compared to AS (Table 1). CSVY’s of 342–365 TCID_50_/cell were obtained in TFDF cultures, corresponding to a >twofold improvement compared to ATF and previous batch and AS cultivations. As both ATF and AS perfusion systems were infected at higher VCC’s, this might indicate the presence of a cell density effect. In terms of VVP, TFDF cultures were comparable to batch cultivations; however, all perfusion systems had an increased STYs, with TFDF cultures showing an increase of >460% compared to batch cultivations.

**Fig. 5:**
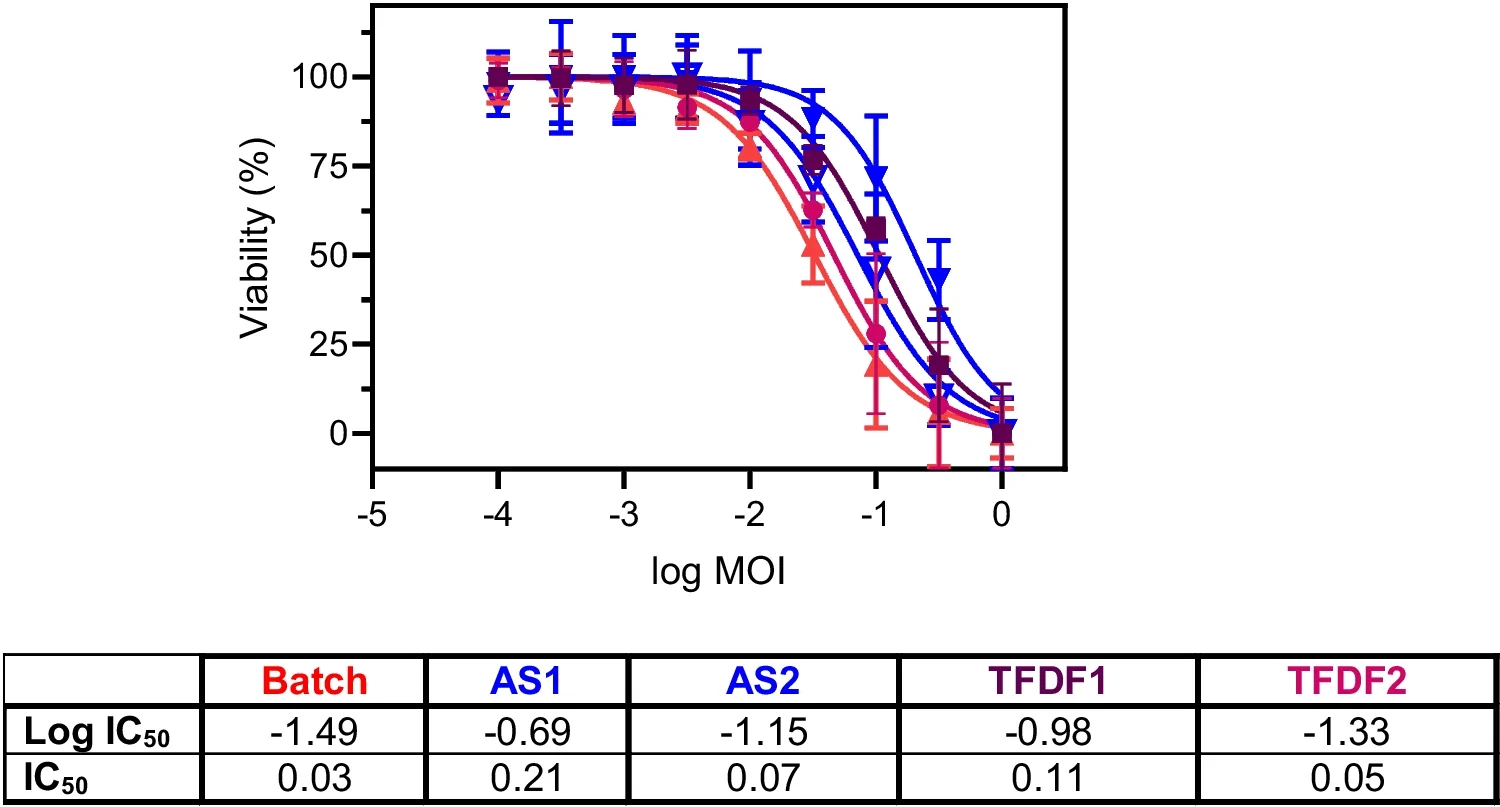
Comparison of oncolytic viral potency values for rVSV-NDV using different production modes and processes. Viabilities of Huh7 cancer cells were determined 48 hpi for crude rVSV-NDV samples generated in batch mode (red triangle), acoustic settler perfusions (AS1, blue full triangle; AS2, hollow blue triangle), and TFDF perfusions (TFDF1, purple square; TFDF2, pink circle). Following non-linear regression analysis, IC_50_ and log IC_50_ values were determined from dose-response curves. All values are reported as the mean of technical triplicates with *n*=3

In a final step, we evaluated whether sporadic formation of syncytia in TFDF mode (Figure S4) had an impact on the oncolytic properties in target Huh7 cells. Crude samples for both TFDF runs were compared to previously produced STR samples using optimized batch processes and AS perfusions. As expected, all samples displayed a similar oncolytic potential in Huh7 cells (Fig. 5). Regardless of the production mode or production system, the produced virus still maintained the ability to induce adequate oncolysis.

### Production of rVSV-GFP in batch mode

**Fig. 6:**
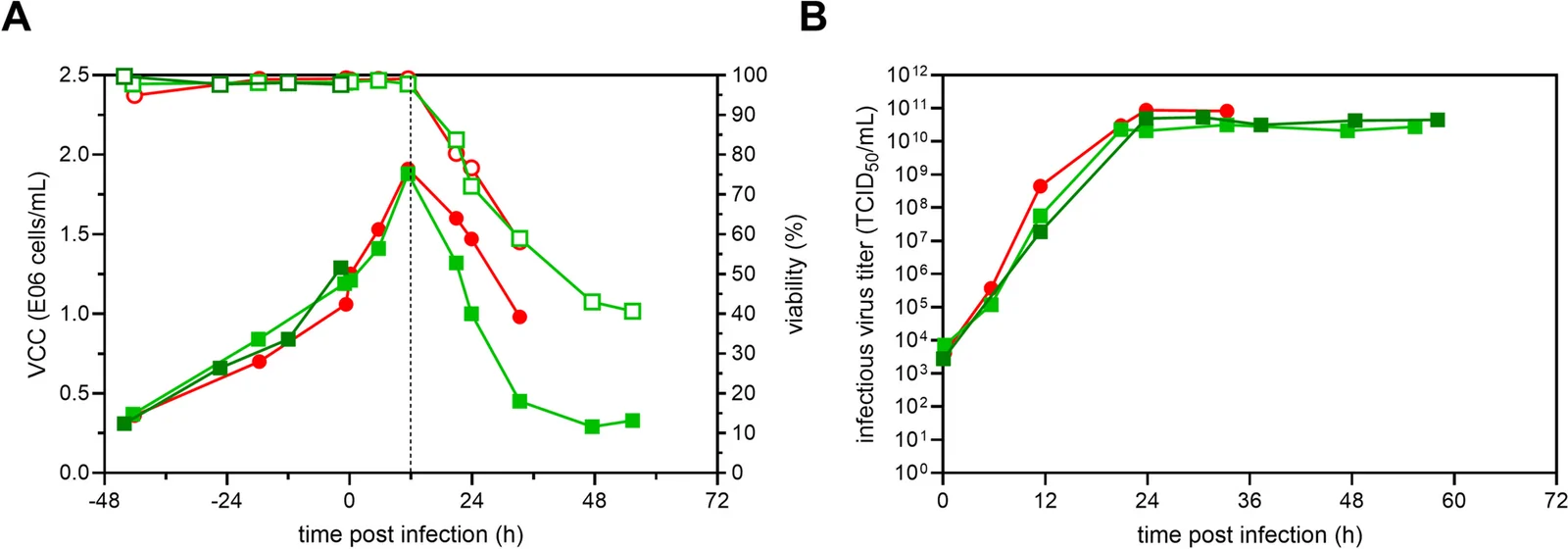
rVSV-GFP production in HEK293-SF cells in batch mode. Three batch production runs were conducted using one shake flask (SF 1, 50 mL WV, red circles), one 1 L STR (STR 1, 700 mL WV, light green squares), and one 3 L STR (STR 2, 2100 mL, dark green squares). Cells were inoculated at 0.3–0.4×10^6^ cells/mL and cultivated to 1.1–1.3×10^6^ cells/mL. Prior to infection, temperature was lowered from 37 to 34 °C. Cells were infected at an MOI of 1E-3. **A** VCC (full symbols) and viability (hollow symbols). **B** Infectious virus titer (TCID_50_/mL)

Initially, a high-yield production process for rVSV-GFP in batch mode in shake flasks (SF) was developed (Fig. 6). Next, the process was transferred to two STRs of different sizes (STR 1, 700 mL WV; STR 2, 2100 mL WV). For this, HEK293-SF cells were inoculated at about 0.3×10^6^ cells/mL and grown to 1.3×10^6^ cells/mL (STR 1 and 2) or 1.1×10^6^ cells/mL (SF) with viabilities above 95% (Fig. 6A). For the three cultivations, very similar cell growth and viability patterns were observed. Cell-specific growth rates ranged between 0.026 and 0.034 1/h (Table 2). As optimized in two recent studies for rVSV-based constructs, cells were infected at an MOI of 1E-3 after a temperature reduction to 34 °C. (Elahi et al. 2019; Gélinas et al. 2019). Maximum VCCs of 1.9×10^6^ cells/mL were reached at 11 hpi for SF and STR 1 cultures, before virus-induced cell death occurred (Fig. 6A, for STR 2 data are not available). Virus production dynamics were very comparable between the three productions (Fig. 6B). Slightly higher maximum infectious virus titers were achieved for production in SF (8.8×10^10^ TCID_50_/mL, 24 hpi) relative to STR 1 (3.2×10^10^ TCID_50_/mL, 33 hpi) and STR 2 (5.3×10^10^ TCID_50_/mL, 31 hpi). Thus, higher CSVY and STY/VVP were obtained for SF production (Table 2). Both STR runs showed high infectious virus titers for more than 36 h indicating a high stability of the virus.

**Table 2:** Comparison of rVSV-GFP production in HEK293-SF cells for different production modes. Cell growth parameters were determined before infection.

	Batch			Perfusion
	STR 1 (1 L)	STR 2 (3 L)	SF	TFDF STR (3 L)
Cell-specific growth rate (1/h)	0.028	0.034	0.026	0.022
Doubling time (h)	24.6	20.7	26.6	31.0
Max. VCC p.i. (10^6^ cells/mL)	1.9	n.d.	1.9	11.3
Max. infectious virus titer (10^10^ TCID_50_/mL)	3.2	5.3	8.8	7.1
CSVY (TCID_50_/cell)	16,865	n.d.	45,948	10,338
STY (10^13^ TCID_50_/L/d)	1.0	1.7	2.8	1.9
VVP (10^13^ TCID_50_/L/d)	1.0	1.7	2.8	0.2
Used medium (L)	0.67	2.10	4.60	13.93

*max*., maximum; *VCC*, viable cell concentration; *p.i*., post infection; *n.d*., not determined. Optimal harvest time point was defined as time point when the maximum infectious virus titer was reached in the supernatant. For the perfusion run, the calculations were based on infectious virus particles of the collected permeate in the harvest (stored at 4 °C) and final harvest

### Production of rVSV-GFP in perfusion mode using a TFDF system

**Fig. 7:**
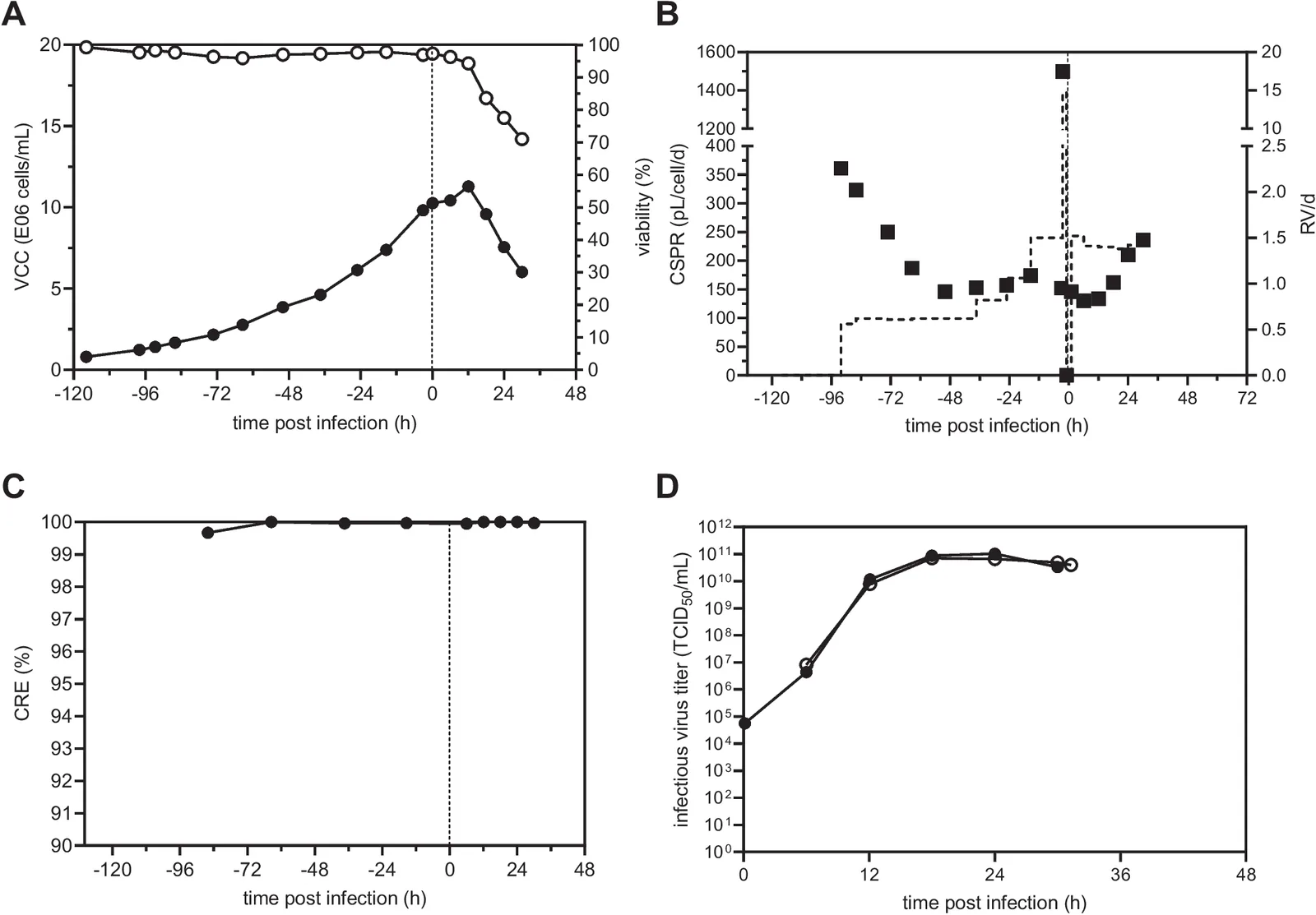
rVSV-GFP production in HEK293-SF cells in perfusion mode using a 3-L STR coupled to a TFDF system. Recirculation was started prior to inoculation. HEK293-SF cells were inoculated at 0.8×10^6^ cells/mL, and perfusion was started after 24 h of batch growth by utilizing a TFDF cartridge (pore size 2–5 μm). The perfusion rate was adjusted manually over time. Prior to infection, temperature was reduced to 34 °C. Cells (10×10^6^ cells/mL) were infected at an MOI of 1E-3, and perfusion was paused for 1 h. **A** Viable cell concentration (full) and viability (empty). **B** Cell-specific perfusion rate (full) and perfusion rate (dashed lined, weight of collected permeate divided by WV). **C** Cell retention efficiency (full) of TFDF membrane during growth and infection. **D** Infectious virus titer (TCID_50_/mL) in the reactor (full symbols) and permeate line (empty symbols) are plotted against the time post infection (h). Last time point shown for the permeate line (31 hpi) refers to the final harvest step

To further support our proof-of-concept study of a TFDF system, we evaluated the production process for rVSV-GFP cultivating HEK293-SF cells in perfusion mode to reach higher VCC and infectious virus titers (Fig. 7). For this, as for the TFDF runs for rVSV-NDV, a 30-cm^2^ TFDF cartridge (pore size 2–5 μm), connected to a Krosflo TFDF system (Repligen), was coupled to the 3 L STR. Recirculation was already started before cell inoculation with a recirculation rate of 1.0 L/min corresponding to a shear rate of 1830 1/s. HEK293-SF cells were inoculated at 0.8×10^6^ cells/mL into the 3 L STR (2100 mL WV) (Fig. 7A). After a batch growth phase for 24 h, perfusion mode was started (Fig. 7B). Cells grew at a slightly lower cell-specific growth rate of 0.022 1/h relative to the batch cultivation in STR 2 (0.028 to 0.034 1/h) (Table 2) during the total cell growth phase, while viabilities remained above 95% (Fig. 7A). The perfusion rate was manually adjusted based on a CSPR of 115 pL/cell/day (Fig. 7B). Due to an initial permeate flow rate of 0.9 mL/min (0.6 RV/day) (Fig. 7B), the actual CSPR was higher at the beginning of the cultivation. Before infection, one RV with fresh medium was exchanged (10–33 mL/min) (Fig. 7B), and temperature was lowered from 37 to 34 °C. Cells were infected at 10.3×10^6^ cells/mL at an MOI of 1E-3. After infection, perfusion was stopped for 1 h and, subsequently, the perfusion rate was kept constant at 1.4 RV/day (Fig. 7B). During the perfusion cultivation, no glucose or glutamine limitation was observed (Figure S6). Moreover, no toxic maximum levels of lactate (22.4 mM) or ammonium (1.4 mM) were found. After infection, cells continued to grow slightly until 11.3×10^6^ cells/mL (12 hpi) with viabilities above 94% (Fig. 7A). Throughout the whole cultivation, the cell retention efficiency of the membrane was maintained above 99.6% (Fig. 7C). A maximum infectious virus titer of 7.1×10^10^ TCID_50_/mL at 18 hpi was detected in the permeate line relative to 10.4×10^10^ TCID_50_/mL at 24 hpi in the bioreactor vessel. Very similar infectious virus titers were observed by comparing the bioreactor vessel and permeate line during the virus production phase (Fig. 7D). On average, the percentage of infectious virus passing through the TFDF membrane was calculated to be 112%. In addition, the infectious virus titer of the final harvest step (4.0×10^10^ TCID_50_/mL) starting at 31 hpi using the same TFDF membrane was similar to the last permeate sample time point of the production phase (4.9×10^10^ TCID_50_/mL, 30 hpi), confirming that all infectious virus particles passed through the membrane even in the final harvest step with a significantly higher permeate flow rate that could cause membrane clogging. Comparing the theoretical maximum values of the amount of infectious virus particles in the permeate line (Figure S7) with the empirical values of the virus in the harvest bulk (collected permeate stored at 4 °C and final harvest, sampled individually) resulted in a recovery of 103.5%, indicating no loss of virus infectivity during storage in the virus production phase. Next, we compared important production coefficients of the batch and perfusion run (Table 2). The perfusion process showed increased STY (1.9-fold (STR 1) and 1.1-fold (STR 2)); however, VVP and CSVY were lower.

## Discussion

The first vaccine based on rVSV, the Ebola virus vaccine Ervebo^®^, has been approved in 2019 (EMA 2019; FDA 2019). Moreover, multiple rVSV-based constructs for vaccine and oncolytic applications are currently undergoing clinical trials. Therefore, the development of a high-yield production platform is essential to meet clinical trial and market demand regardless of the intended use. Compared to traditional vector platforms, induction of syncytia by rVSV-NDV introduces additional challenges to manufacturing processes. In this proof-of-concept study, we evaluated the versatility of the TFDF system for intensified high-cell density production for two different rVSV-based constructs (rVSV-NDV and rVSV-GFP) to allow continuous virus harvest and clarification.

### rVSV-NDV production using mATF

Product retention of hollow-fiber membranes is a well-known challenge of ATF-based cell culture productions, preventing continuous harvest of, e.g., viral particles (Genzel et al. 2014; Hadpe et al. 2017; Nikolay et al. 2020; Nikolay et al. 2018; Tona et al. 2023; Vázquez-Ramírez et al. 2019). To evaluate the applicability of hollow-fiber membranes for the production of fusogenic oncolytic viruses forming large muti-nucleated syncytia, a commercially available 0.65-μm mPES membrane with a lumen size bigger than previously observed syncytia (120–140 μm) was selected, connected to a mATF, and characterized for virus retention. Impact of the membrane material on product retention is still not fully elaborated. While some studies found a more pronounced retention rate of antibodies using polysulfone (PS) membranes compared to polyethersulfone (PES) and mPES due to the higher negative charge densities (Su et al. 2021), others reported more favorable physiochemical and structural properties (open pore structure, high porosity) of PS membranes enabling virus harvest of yellow fever virus particles (~50 nm) even at low cut-offs of 0.34 μm (Nikolay et al. 2020). Beside membrane material, pore size plays a critical role regarding product retention. Surprisingly, membranes with higher cut-offs, e.g., 0.65 μm often display higher product retention rates compared to lower cut-offs, e.g., 0.2 μm (Nikolay et al. 2020; Su et al. 2021; Vázquez-Ramírez et al. 2019). More pronounced heterogeneous pore distributions for larger cut-offs can increase the susceptibility of fouling as variation in filtrate flux along the membrane makes larger pores prone to deposition of particles and concentration polarization (Nikolay et al. 2020). Moreover small-size cell debris (0.2–0.5 μm), caused by virus induced cell lysis, can enter larger pores causing membrane clogging and product retention, but can be rejected by smaller pores (Su et al. 2021). Usage of a 0.2-μm PES hollow fiber membrane for a rVSV-NDV ATF production using HEK293 cells resulted in a complete membrane blocking at 18 hpi, after first syncytia formation was observed (data not shown). Therefore, the choice of membrane was primarily based on internal fiber lumen rather than pore size or material. A higher flow rate inside the hollow fibers (1.5 L/min) was chosen compared to the initial ATF run using a 0.2-μm PES membrane (0.8 L/min) to increase the backflush over the membrane and prevent or hamper the formation of syncytia due to increased shear stress. Surprisingly, the resulting shear rate of 5490 1/s neither impacted cell growth, as high VCCs of 44.5×10^6^ cells/mL with high cell-specific growth rates of 0.031 1/h (Table 1) were reached, nor did it prevent the formation of syncytia (Figure S4). However, formed syncytia were smaller (up to 75-μm diameter) as previously observed with an acoustic settler (up to 140 μm (Göbel et al. 2023a)), allowing entrance into the larger fibers, which likely prevented complete blockage. Nevertheless, combination of high hollow fiber flow rates with low permeate flux rates of 1.91 L/h/m^2^ did not prevent rVSV-NDV retention by the membrane. Already at 18 hpi, 97% of all infectious virus was retained, further underlining the challenge of using hollow fiber membranes for continuous harvest of viral particles.

### rVSV-NDV production using TFDF

As a proof-of-concept, we set out to utilize the TFDF system as a cell retention device for perfusion and subsequent continuous harvest filtration. Performance was characterized by cell growth, TMP, and quantification of bioreactor and permeate turbidities, as well as virus permeability. The use of the TFDF system allowed BHK-21 cells to achieve high VCCs, with similar growth behavior compared to the mATF system despite the drastically lower shear stress there. Due to the lower CSVY obtained at very high VCCs in the previous experiment, the targeted cell concentration for infection was lowered drastically. The metabolic uptake rates for all perfusion cultures were slightly increased compared to those previously reported for batch cultivations ((Göbel et al. 2023a), Table 1). Increased shear stress, particularly in ATF and TFF systems, has been identified as one cause of increased substrate uptake rates (Zhan et al. 2020). Control of the perfusion rate via a capacitance probe did not improve overall process performance, but robustly maintained stable CSPR values over the entire growth phase (Fig. 3), reducing medium consumption by 15%. Compared to the manual perfusion control for TFDF1, where CSPR values were higher due to partial overfeeding, glucose levels were not stably maintained and fell below 5 mM prior to infection (Figure S2). The slightly increased growth rate of TFDF2 most likely resulted in an increased uptake of glucose and thus increased lactate formation. To support even higher VCCs, the set point of the CSPR should be increased for future capacitance-controlled runs.

The combination of DF with tangential cross filtration provides several benefits such as shearing of the membrane surface, minimizing deposition of particles within the filter, while simultaneously allowing some particles to be captured within channels of the DF without blocking the liquid flow through that same channel (Williams et al. 2020). Low TMP values (below 0.3 psi) for both TFDF runs, even after virus infection, high cell retention efficiencies (>99%), and a low turbidity in the permeate (>95% reduction) indicated minimal particle breakthrough. Previous studies already demonstrated the applicability of the TFDF system for continuous harvest of AAV and LV (Mendes et al. 2022; Tona et al. 2023; Tran and Kamen 2022). As expected, we also achieved equal concentrations of infectious virus in both the bioreactor and permeate sample, taken at the same time. Calculated percentages of infectious virus passing through the membrane above 100% for both runs are not possible and were only achieved as the measured titer in the permeate sample was higher than the bioreactor sample. While comparison to theoretical yields only resulted in a total recovery of 78%, this is most likely due to partial losses of functionality while storing the harvest bulks at RT, as well as the quite large error of the TCID_50_ assay itself (±0.3 log (Göbel et al. 2022b)). Stabilizing effects of sucrose on proteins and enveloped viral vectors are well known (Croyle et al. 2001; Cruz et al. 2006; Evans et al. 2004); however, it acts mainly as a cryoprotectant or is only a small part of a complex storage formulation. Therefore, premature addition of sucrose was probably not sufficient to prevent degradation, and direct cooling of the harvest bulk at 4 °C should be preferred.

In terms of virus production, our intensified TFDF processes achieved the highest reported infectious virus titers of 5.6–7.5x×10^9^ TCID_50_/mL in the permeate so far. Compared to optimized batch processes, VCCs were increased by five- to six-fold, but infectious virus titers were even more than 11-fold higher. Moreover, CSVYs were improved by twofold, STY by 460% (5.6-fold), and similar VVPs were reached. Lastly, the TFDF runs were compared to other perfusion cultivations using the same cell line but different cell retention devices. Compared to AS and ATF perfusions, maximum titers reached in TFDF systems were >threefold and >1.5-fold higher. This was also reflected in terms of VVP and STY, which were always more than twofold higher for TFDF runs. Surprisingly, CSVYs strongly decreased with increasing VCCs for the respective systems. For the ATF cultivation, where the highest VCC of 44.5×10^6^ cells/mL was reached, the lowest CSVY was obtained, clearly indicating the presence of a “high cell density effect” (Bock et al. 2011; Nadeau and Kamen 2003). One major reason for this effect is typically the scarcity in nutrients or the accumulation of inhibitory ammonium and glucose. One study showed that ammonium and lactate concentrations at 2–3 mM and above 20–30 mM, respectively, can have negative effects on virus productivity and cell growth (Schneider et al. 1996). However, neither a nutrient limitation nor an accumulation of lactate and ammonium to excessively high concentrations was observed for any run ((Göbel et al. 2023a), Figure S2, data not shown for ATF). This suggests that other reasons, including the limitation or accumulation of non-monitored metabolites or unknown cellular factors may play a role and are subject to further investigation. Another reason could be the formation of syncytia, which was observed to occur to differential extends, depending on the system used (Figure S4). Virus replication is possibly more efficient if the cells do not fuse, as we have observed the production of higher titers in oncolytic applications when a non-fusogenic VSV is used, despite similar levels of oncolysis (Abdullahi et al. 2018); however, whether or not this is also true in suspension culture systems which is not entirely clear and would warrant further investigation. Regardless, the direct comparison of the three perfusion systems should be considered very carefully, as various production parameters were different. For a fair comparison, re-evaluation should include the same bioreactor set-up and similar infection cell concentrations. For our proof-of-concept study this was, however, out of scope.

Fusogenic oncolytic viruses and the formation of syncytia introduce novel challenges for process controls and scale-up of manufacturing processes. We hypothesize that fusion of cells is dependent on three factors: high VCC, low shear stress, and long cell-to-cell contact time. Combination of all three factors most likely facilitates the formation of large multi-nucleated syncytia. Production in batch mode is associated with low VCC (up to 3.2×10^6^ cells/mL), low shear stress, and short cell-to-cell contact times and does not lead to the formation of syncytia. Productions using ATF or TFDF systems allow for high VCCs (up to 44.5×10^6^ cells/mL); however, shear rates are drastically increased (up to 5490 1/s), and cell-to-cell contact times are short, leading to the formation of small sized syncytia. Retention systems such as AS, combine high VCCs (up to 29.7×10^6^ cells/mL), low shear rates (~340 1/s (Gränicher et al. 2020)), and long cell-to-cell contact times within the acoustic field and recirculation loop (3–12 min (Gränicher et al. 2020)), facilitating the formation of large multi-nucleated syncytia. Increased cell-to-cell contact by induction of aggregation by CaCl_2_ supplementation at low VCC in batch mode did not result in the formation of syncytia, highlighting the complex interplay of all three factors (Göbel et al. 2022a). Future studies investigating the actual cause of syncytia formation in suspension cultures could be considered to better control their formation. However, whether or not syncytia are formed in suspension cultures during production is independent of the inherent fusogenicity of the virus, as the fusion proteins are encoded within its genome and need to be expressed in order to carry out an infection (Abdullahi et al. 2018). Nonetheless, a potency assay was carried out to assess potential effects of mode of production, as well as formation or non-formation of syncytia during production, on the ability of the virus to induce oncolysis. As expected, oncolytic potency was not affected by production mode or occurrence of syncytia (Fig. 5).

### rVSV-GFP production in batch and perfusion mode

To take our proof-of-concept study one step further, we also wanted to evaluate the TFDF performance for a high-yield virus production process for rVSV-based vectors for a possible application as vaccine using the model vector rVSV-GFP. Process intensification using perfusion mode and the TFDF module led to a sixfold higher VCC of 11.3×10^6^ cells/mL with an up to 1.9-fold higher STY compared to the STR 1 process in batch mode, allowing for a smaller footprint of the bioreactor. However, a more than 3.3-fold lower VVP and 1.6-fold reduced CSVY were observed compared to the STR batch process. As described before, this decline is most likely due to the “high cell density effect.” As neither limitation of monitored nutrients, nor accumulation of inhibitory byproducts was observed, it is clear that there is certainly room for optimization for this perfusion process, hopefully targeting even higher VCCs. For now, this perfusion run was a proof-of-concept run only to evaluate the TFDF system. Testing different feeding schemes (Vázquez-Ramírez et al. 2019), media compositions (Göbel et al. 2023a), or additives could be envisaged as next steps.

Using the TFDF module, we were able to directly harvest rVSV-GFP particles with a simultaneous clarification by depth filtration with a full recovery. Our proof-of-concept study together with the data on LV (Tona et al. 2023; Tran and Kamen 2022) and AAV (Mendes et al. 2022) seems to indicate that TFDF for continuous virus harvest in perfusion will play a big role in next generation processes and might be applicable for other viruses as well. Moreover, all HEK293-SF cells were retained inside the bioreactor enabling full production capacity. Perfusion cultivation with the TFDF module showed a slightly lower cell-specific growth rate (0.022 1/h) relative to the batch production (0.034 to 0.028 1/h), indicating that there is also room for improvement here and a need for further optimization. As previously discussed, cooling the harvest bulk to 4 °C increases virus stability. Indeed, we found a recovery of 103.5% in the final harvest bulk.

Overall, the TFDF module showed very good performance as a perfusion system for our tested rVSV-based vectors and cell lines. In addition, the continuous virus harvest, together with the clarification through the TFDF module in one step can simplify process operations and help to develop an integrated, scalable (up to 2000 L), and economical process for the future.

## Supplementary information

ESM 1 (PDF 609 kb)

## Data Availability

Data available in article supplementary material. Additional data is available on request from the authors. The data that support the findings of this study are available from the corresponding author, Yvonne Genzel, upon reasonable request.

## References

[CR1] Abdullahi S, Jäkel M, Behrend SJ, Steiger K, Topping G, Krabbe T, Colombo A, Sandig V, Schiergens TS, Thasler WE, Werner J, Lichtenthaler SF, Schmid RM, Ebert O, Altomonte J (2018) A novel chimeric oncolytic virus vector for improved safety and efficacy as a platform for the treatment of hepatocellular carcinoma. J Virol 92(23). 10.1128/jvi.01386-18 10.1128/JVI.01386-18 PMC6232488 30232179

[CR2] Aunins JG (2003) Viral vaccine production in cell culture. In: Spier RE (ed) Encyclopedia of cell technology. 10.1002/0471250570.spi105

[CR3] Bock A, Schulze-Horsel J, Schwarzer J, Rapp E, Genzel Y, Reichl U (2011) High-density microcarrier cell cultures for influenza virus production. Biotechnol Progr 27(1):241–250. 10.1002/btpr.539 10.1002/btpr.539 21312371

[CR4] Brown KS, Safronetz D, Marzi A, Ebihara H, Feldmann H (2011) Vesicular stomatitis virus-based vaccine protects hamsters against lethal challenge with Andes virus. J Virol 85(23):12781–12791. 10.1128/jvi.00794-11 21917979 10.1128/JVI.00794-11 PMC3209372

[CR5] Cobleigh MA, Buonocore L, Uprichard SL, Rose JK, Robek MD (2010) A vesicular stomatitis virus-based hepatitis B virus vaccine vector provides protection against challenge in a single dose. J Virol 84(15):7513–7522. 10.1128/jvi.00200-10 20504927 10.1128/JVI.00200-10 PMC2897621

[CR6] Coronel J, Behrendt I, Bürgin T, Anderlei T, Sandig V, Reichl U, Genzel Y (2019) Influenza A virus production in a single-use orbital shaken bioreactor with ATF or TFF perfusion systems. Vaccine 37(47):7011–7018. 10.1016/j.vaccine.2019.06.005 31266669 10.1016/j.vaccine.2019.06.005

[CR7] Coronel J, Gränicher G, Sandig V, Noll T, Genzel Y, Reichl U (2020) Application of an inclined settler for cell culture-based influenza A virus production in perfusion mode. Front Bioeng Biotechnol 8:672–672. 10.3389/fbioe.2020.00672 32714908 10.3389/fbioe.2020.00672 PMC7343718

[CR8] Croyle MA, Cheng X, Wilson JM (2001) Development of formulations that enhance physical stability of viral vectors for gene therapy. Gene Ther 8(17):1281–1290. 10.1038/sj.gt.3301527 11571564 10.1038/sj.gt.3301527

[CR9] Cruz PE, Silva AC, Roldão A, Carmo M, Carrondo MJ, Alves PM (2006) Screening of novel excipients for improving the stability of retroviral and adenoviral vectors. Biotechnol Prog 22(2):568–576. 10.1021/bp050294y 16599578 10.1021/bp050294y

[CR10] DeBuysscher BL, Scott D, Thomas T, Feldmann H, Prescott J (2016) Peri-exposure protection against Nipah virus disease using a single-dose recombinant vesicular stomatitis virus-based vaccine. NPJ Vaccines 1:16002. 10.1038/npjvaccines.2016.2 28706736 10.1038/npjvaccines.2016.2 PMC5505655

[CR11] Eccles R (2021) Why is temperature sensitivity important for the success of common respiratory viruses? Rev Med Virol 31(1):1–8. 10.1002/rmv.2153 32776651 10.1002/rmv.2153 PMC7435572

[CR12] Elahi SM, Shen CF, Gilbert R (2019) Optimization of production of vesicular stomatitis virus (VSV) in suspension serum-free culture medium at high cell density. J Biotechnol 289:144–149 30508556 10.1016/j.jbiotec.2018.11.023

[CR13] EMA (2019) Ervebo: Ebola vaccine (rVSVΔG-ZEBOV-GP, live). European Medicines Agency. https://www.ema.europa.eu/en/medicines/human/EPAR/ervebo

[CR14] Emanuel J, Callison J, Dowd KA, Pierson TC, Feldmann H, Marzi A (2018) A VSV-based Zika virus vaccine protects mice from lethal challenge. Sci Rep 8(1):11043. 10.1038/s41598-018-29401-x 30038228 10.1038/s41598-018-29401-x PMC6056530

[CR15] Evans RK, Nawrocki DK, Isopi LA, Williams DM, Casimiro DR, Chin S, Chen M, Zhu DM, Shiver JW, Volkin DB (2004) Development of stable liquid formulations for adenovirus-based vaccines. J Pharm Sci 93(10):2458–2475. 10.1002/jps.20157 15349956 10.1002/jps.20157

[CR16] FDA (2019) First FDA-approved vaccine for the prevention of Ebola virus disease: marking a critical milestone for public health. FDA. https://www.fda.gov/news-events/press-announcements/first-fda-approved-vaccine-preventionebola-virus-disease-marking-critical-milestone-public-health

[CR17] Geisbert TW, Jones S, Fritz EA, Shurtleff AC, Geisbert JB, Liebscher R, Grolla A, Ströher U, Fernando L, Daddario KM, Guttieri MC, Mothé BR, Larsen T, Hensley LE, Jahrling PB, Feldmann H (2005) Development of a new vaccine for the prevention of Lassa fever. PLoS Med 2(6):e183. 10.1371/journal.pmed.0020183 15971954 10.1371/journal.pmed.0020183 PMC1160587

[CR18] Gélinas J-F, Azizi H, Kiesslich S, Lanthier S, Perdersen J, Chahal PS, Ansorge S, Kobinger G, Gilbert R, Kamen AA (2019) Production of rVSV-ZEBOV in serum-free suspension culture of HEK 293SF cells. Vaccine 37(44):6624–6632. 10.1016/j.vaccine.2019.09.044 31548015 10.1016/j.vaccine.2019.09.044

[CR19] Genzel Y, Dietzsch C, Rapp E, Schwarzer J, Reichl U (2010) MDCK and Vero cells for influenza virus vaccine production: a one-to-one comparison up to lab-scale bioreactor cultivation. Appl Microbiol Biotechnol 88(2):461–475. 10.1007/s00253-010-2742-9 20617311 10.1007/s00253-010-2742-9 PMC7080112

[CR20] Genzel Y, Vogel T, Buck J, Behrendt I, Ramirez DV, Schiedner G, Jordan I, Reichl U (2014) High cell density cultivations by alternating tangential flow (ATF) perfusion for influenza A virus production using suspension cells. Vaccine 32(24):2770–2781. 10.1016/j.vaccine.2014.02.016 24583003 10.1016/j.vaccine.2014.02.016

[CR21] Göbel S, Jaén KE, Dorn M, Neumeyer V, Jordan I, Sandig V, Reichl U, Altomonte J, Genzel Y (2023a) Process intensification strategies towards cell culture-based high-yield production of a fusogenic oncolytic virus. Biotechnol Bioeng. 10.1002/bit.28353 10.1002/bit.28353 36779302

[CR22] Göbel S, Jaén KE, Fernandes RP, Reiter M, Altomonte J, Reichl U, Genzel Y (2023b) Characterization of a quail suspension cell line for production of a fusogenic oncolytic virus. Biotechnol Bioeng. 10.1002/bit.28530 10.1002/bit.28530 37584190

[CR23] Göbel S, Kortum F, Chavez KJ, Jordan I, Sandig V, Reichl U, Altomonte J, Genzel Y (2022a) Cell-line screening and process development for a fusogenic oncolytic virus in small-scale suspension cultures. Appl Microbiol Biotechnol 106(13-16):4945–4961. 10.1007/s00253-022-12027-5 35767011 10.1007/s00253-022-12027-5 PMC9329169

[CR24] Göbel S, Pelz L, Reichl U, Genzel Y (2022b) Chapter 5 Upstream processing for viral vaccines– Process intensification. In: Amine Kamen LC (ed) Bioprocessing of viral vaccines, vol 1. Taylor & Francis Group, https://www.routledge.com/Bioprocessing-of-Viral-Vaccines/Kamen-Cervera/p/book/9781032132112, pp 79–137

[CR25] Gränicher G, Babakhani M, Göbel S, Jordan I, Marichal-Gallardo P, Genzel Y, Reichl U (2021a) A high cell density perfusion process for modified Vaccinia virus Ankara production: process integration with inline DNA digestion and cost analysis. Biotechnol Bioeng. 10.1002/bit.27937 10.1002/bit.27937 34506646

[CR26] Gränicher G, Coronel J, Trampler F, Jordan I, Genzel Y, Reichl U (2020) Performance of an acoustic settler versus a hollow fiber–based ATF technology for influenza virus production in perfusion. Appl Microbiol Biotechnol 104(11):4877–4888. 10.1007/s00253-020-10596-x 32291490 10.1007/s00253-020-10596-x PMC7228903

[CR27] Gränicher G, Tapia F, Behrendt I, Jordan I, Genzel Y, Reichl U (2021b) Production of modified Vaccinia Ankara virus by intensified cell cultures: a comparison of platform technologies for viral vector production. Biotechnol J 16(1):e2000024. 10.1002/biot.202000024 32762152 10.1002/biot.202000024 PMC7435511

[CR28] Hadpe SR, Sharma AK, Mohite VV, Rathore AS (2017) ATF for cell culture harvest clarification: mechanistic modelling and comparison with TFF. J Chem Technol Biotechnol 92(4):732–740. 10.1002/jctb.5165

[CR29] Hein MD, Chawla A, Cattaneo M, Kupke SY, Genzel Y, Reichl U (2021) Cell culture-based production of defective interfering influenza A virus particles in perfusion mode using an alternating tangential flow filtration system. bioRxiv 2021.06.07.446880. 10.1101/2021.06.07.446880 10.1007/s00253-021-11561-y PMC8437742 34519855

[CR30] Jones SM, Feldmann H, Ströher U, Geisbert JB, Fernando L, Grolla A, Klenk HD, Sullivan NJ, Volchkov VE, Fritz EA, Daddario KM, Hensley LE, Jahrling PB, Geisbert TW (2005) Live attenuated recombinant vaccine protects nonhuman primates against Ebola and Marburg viruses. Nat Med 11(7):786–790. 10.1038/nm1258 15937495 10.1038/nm1258

[CR31] Kahn JS, Roberts A, Weibel C, Buonocore L, Rose JK (2001) Replication-competent or attenuated, nonpropagating vesicular stomatitis viruses expressing respiratory syncytial virus (RSV) antigens protect mice against RSV challenge. J Virol 75(22):11079–11087. 10.1128/jvi.75.22.11079-11087.2001 11602747 10.1128/JVI.75.22.11079-11087.2001 PMC114687

[CR32] Krabbe T, Marek J, Groll T, Steiger K, Schmid RM, Krackhardt AM, Altomonte J (2021) Adoptive T cell therapy is complemented by oncolytic virotherapy with fusogenic VSV-NDV in combination treatment of murine melanoma. Cancers 13(5):1044 33801359 10.3390/cancers13051044 PMC7958625

[CR33] Lauretti F, Chattopadhyay A, de Oliveira França RF, Castro-Jorge L, Rose J, Fonseca BA (2016) Recombinant vesicular stomatitis virus-based dengue-2 vaccine candidate induces humoral response and protects mice against lethal infection. Human Vaccines Immunother 12(9):2327–2333. 10.1080/21645515.2016.1183857 10.1080/21645515.2016.1183857 PMC5027729 27185081

[CR34] Liao JB, Publicover J, Rose JK, DiMaio D (2008) Single-dose, therapeutic vaccination of mice with vesicular stomatitis virus expressing human papillomavirus type 16 E7 protein. Clin Vaccine Immunol: CVI 15(5):817–824. 10.1128/cvi.00343-07 18337377 10.1128/CVI.00343-07 PMC2394842

[CR35] Manceur AP, Kim H, Misic V, Andreev N, Dorion-Thibaudeau J, Lanthier S, Bernier A, Tremblay S, Gélinas A-M, Broussau S, Gilbert R, Ansorge S (2017) Scalable lentiviral vector production using stable HEK293SF producer cell lines. Hum Gene Ther Methods 28(6):330–339. 10.1089/hgtb.2017.086 28826344 10.1089/hgtb.2017.086 PMC5734158

[CR36] Mendes JP, Fernandes B, Pineda E, Kudugunti S, Bransby M, Gantier R, Peixoto C, Alves PM, Roldão A, Silva RJS (2022) AAV process intensification by perfusion bioreaction and integrated clarification. Front Bioeng Biotechnol 10:1020174. 10.3389/fbioe.2022.1020174 36420444 10.3389/fbioe.2022.1020174 PMC9676353

[CR37] Mendonça SA, Lorincz R, Boucher P, Curiel DT (2021) Adenoviral vector vaccine platforms in the SARS-CoV-2 pandemic. NPJ Vaccines 6(1):97. 10.1038/s41541-021-00356-x 34354082 10.1038/s41541-021-00356-x PMC8342436

[CR38] Moleirinho MG, Silva RJS, Alves PM, Carrondo MJT, Peixoto C (2020) Current challenges in biotherapeutic particles manufacturing. Expert Opinion Biol Ther 20(5):451–465. 10.1080/14712598.2020.1693541 10.1080/14712598.2020.1693541 31773998

[CR39] Nadeau I, Kamen A (2003) Production of adenovirus vector for gene therapy. Biotechnol Adv 20(7-8):475–489 14550017 10.1016/s0734-9750(02)00030-7

[CR40] Nikolay A, de Grooth J, Genzel Y, Wood JA, Reichl U (2020) Virus harvesting in perfusion culture: choosing the right type of hollow fiber membrane. Biotechnol Bioeng 117(10):3040–3052. 10.1002/bit.27470 32568408 10.1002/bit.27470

[CR41] Nikolay A, Léon A, Schwamborn K, Genzel Y, Reichl U (2018) Process intensification of EB66® cell cultivations leads to high-yield yellow fever and Zika virus production. Appl Microbiol Biotechnol 102(20):8725–8737. 10.1007/s00253-018-9275-z 30091043 10.1007/s00253-018-9275-z PMC6153634

[CR42] Palin A, Chattopadhyay A, Park S, Delmas G, Suresh R, Senina S, Perlin DS, Rose JK (2007) An optimized vaccine vector based on recombinant vesicular stomatitis virus gives high-level, long-term protection against Yersinia pestis challenge. Vaccine 25(4):741–750. 10.1016/j.vaccine.2006.08.010 16959385 10.1016/j.vaccine.2006.08.010

[CR43] Pelz L, Göbel S, Chavez K, Reichl U, Genzel Y (2022) Chapter 5 Upstream processing for viral vaccines—general aspects. In: Amine Kamen LC (ed) Bioprocessing of viral vaccines, vol 1. Taylor & Francis Group, https://www.routledge.com/Bioprocessing-of-Viral-Vaccines/Kamen-Cervera/p/book/9781032132112, pp 79–137

[CR44] Roberts A, Kretzschmar E, Perkins AS, Forman J, Price R, Buonocore L, Kawaoka Y, Rose JK (1998) Vaccination with a recombinant vesicular stomatitis virus expressing an influenza virus hemagglutinin provides complete protection from influenza virus challenge. J Virol 72(6):4704–4711. 10.1128/jvi.72.6.4704-4711.1998 9573234 10.1128/jvi.72.6.4704-4711.1998 PMC109996

[CR45] Schneider M, Marison IW, von Stockar U (1996) The importance of ammonia in mammalian cell culture. J Biotechnol 46(3):161–185. 10.1016/0168-1656(95)00196-4 8672289 10.1016/0168-1656(95)00196-4

[CR46] Su Y, Wei Z, Miao Y, Sun L, Shen Y, Tang Z, Li L, Quan Y, Yu H, Wang W-C, Zhou W, Tian J (2021) Optimized process operations reduce product retention and column clogging in ATF-based perfusion cell cultures. Appl Microbiol Biotechnol 105(24):9125–9136. 10.1007/s00253-021-11662-8 34811605 10.1007/s00253-021-11662-8

[CR47] Suder E, Furuyama W, Feldmann H, Marzi A, de Wit E (2018) The vesicular stomatitis virus-based Ebola virus vaccine: from concept to clinical trials. Human Vaccines Immunother 14(9):2107–2113. 10.1080/21645515.2018.1473698 10.1080/21645515.2018.1473698 PMC6183239 29757706

[CR48] Tona RM, Shah R, Middaugh K, Steve J, Marques J, Roszell BR, Jung C (2023) Process intensification for lentiviral vector manufacturing using tangential flow depth filtration. Mol Ther- Methods Clinic Dev 29:93–107. 10.1016/j.omtm.2023.02.017 10.1016/j.omtm.2023.02.017 PMC10041461 36994313

[CR49] Tran MY, Kamen AA (2022) Production of lentiviral vectors using a HEK-293 producer cell line and advanced perfusion processing. Front Bioeng Biotechnol 10:887716. 10.3389/fbioe.2022.887716 35774066 10.3389/fbioe.2022.887716 PMC9237754

[CR50] Ura T, Okuda K, Shimada M (2014) Developments in viral vector-based vaccines. Vaccines (Basel) 2(3):624–641. 10.3390/vaccines2030624 26344749 10.3390/vaccines2030624 PMC4494222

[CR51] Ura T, Yamashita A, Mizuki N, Okuda K, Shimada M (2021) New vaccine production platforms used in developing SARS-CoV-2 vaccine candidates. Vaccine 39(2):197–201. 10.1016/j.vaccine.2020.11.054 33279318 10.1016/j.vaccine.2020.11.054 PMC7685034

[CR52] van den Pol AN, Mao G, Chattopadhyay A, Rose JK, Davis JN (2017) Chikungunya, influenza, Nipah, and Semliki forest chimeric viruses with vesicular stomatitis virus: actions in the brain. J Virol 91(6). 10.1128/jvi.02154-16 10.1128/JVI.02154-16 PMC5331823 28077641

[CR53] Vázquez-Ramírez D, Jordan I, Sandig V, Genzel Y, Reichl U (2019) High titer MVA and influenza A virus production using a hybrid fed-batch/perfusion strategy with an ATF system. Appl Microbiol Biotechnol 103(7):3025–3035 30796494 10.1007/s00253-019-09694-2 PMC6447503

[CR54] Williams T., Goodyear O., Davies L., Knevelman C., Bransby M., Mitrophanous K., J. M (2020) Lentiviral vector manufacturing process enhancement utilizing TFDF™ technology. Cell Gene Ther Insights;6:455–467 doi:10.18609/cgti.2020.053

[CR55] Wu Y, Bissinger T, Genzel Y, Liu X, Reichl U, Tan W-S (2021) High cell density perfusion process for high yield of influenza A virus production using MDCK suspension cells. Appl Microbiol Biotechnol 105(4):1421–1434. 10.1007/s00253-020-11050-8 33515287 10.1007/s00253-020-11050-8 PMC7847233

[CR56] Yang Z, Paes B, Fulber JPC, Tran MY, Farnós O, Kamen AA (2023) Development of an integrated continuous manufacturing process for the rVSV-Vectored SARS-CoV-2 candidate vaccine. Vaccines (Basel) 11(4). 10.3390/vaccines11040841 10.3390/vaccines11040841 PMC10143285 37112753

[CR57] Yue J, Liu Y, Zhao M, Bi X, Li G, Liang W (2023) The R&D landscape for infectious disease vaccines. Nat Rev Drug Discov. 10.1038/d41573-023-00119-4 10.1038/d41573-023-00119-4 37474662

[CR58] Zhan C, Bidkhori G, Schwarz H, Malm M, Mebrahtu A, Field R, Sellick C, Hatton D, Varley P, Mardinoglu A, Rockberg J, Chotteau V (2020) Low shear stress increases recombinant protein production and high shear stress increases apoptosis in human cells. iScience 23(11):101653. 10.1016/j.isci.2020.101653 33145483 10.1016/j.isci.2020.101653 PMC7593556

[CR59] Zhang Y, Nagalo BM (2022) Immunovirotherapy based on recombinant vesicular stomatitis virus: where are we? Front Immunol 13. 10.3389/fimmu.2022.898631 10.3389/fimmu.2022.898631 PMC9273848 35837384

