# Supplementary material for: Production of recombinant vesicular stomatitis virus-based vectors by tangential flow depth filtration
Source: Appl Microbiol Biotechnol. 2024 Feb 27;108:240. doi: 10.1007/s00253-024-13078-6 (PMC10899354; doi:10.1007/s00253-024-13078-6)
Supplement: Supplementary file 1 [file 253_2024_13078_MOESM1_ESM.pdf]

Journal: Applied Microbiology and Biotechnology

Production of recombinant vesicular stomatitis virus-based vectors by tangential flow depth filtration

Sven Göbel[¶1], Lars Pelz[¶1], Cristina A. T. Silva[2], Béla Brühlmann[3], Charles Hill[3], Jennifer Altomonte[4], Amine Kamen[5], Udo Reichl[1,6], Yvonne Genzel[1*]

[1]Bioprocess Engineering, Max Planck Institute for Dynamics of Complex Technical Systems, Sandtorstr. 1, 39106 Magdeburg, Germany

[2]Department of Chemical Engineering,Polytechnique Montréal, Montréal,Québec, Canada

[3]Repligen, Waltham, MA, United States

[4]Department of Internal Medicine II, Klinikum rechts der Isar, Technische Universität München, Germany

[5]Department of Bioengineering, McGill University, Montréal, Québec, Canada

[6]Chair for Bioprocess Engineering, Otto-von-Guericke-University Magdeburg, Universitätsplatz 2, 39106 Magdeburg, Germany

[¶] These authors contributed equally to this work.

*Corresponding author:
Yvonne Genzel
Email address: genzel@mpi-magdeburg.mpg.de
Telephone:+49 391 6110 257

Table S1 Final bioreactor harvest using C1-DF-C2 process for two perfusion mode productions of rVSV-NDV.

Mode of operation	permeate flow rate (mL/min)		feed flow rate (ml/min)		inlet flow rate (L/min)		harvest volume (mL)	
	TFDF1	TFDF2	TFDF1	TFDF2	TFDF1	TFDF2	TFDF1	TFDF2
**C1**	35	20	0	0	2	2.5	-571	-570
**DF**	0	0	43	32	2	2.5	+1000	+530
**C2**	35	20-30	0	0	2	2.6	-529	-990

All flow rates and volumes of the individual operations steps are listed for TFDF1 and TFDF2 for rVSV-NDV. – represents the withdrawal of liquid through the TFDF membrane, + the addition of liquid to the bioreactor vessel. C1: concentration 1, DF: diafiltration, C2: concentration 2. For TFDF1 PBS was used for the DF step, for TFDF2 medium.

Table S2 Final bioreactor harvest using C1-DF-C2 process for perfusion mode production of rVSV-GFP.

Mode of operation	permeate flow rate (mL/min)	feed flow rate (ml/min)	inlet flow rate (L/min)	harvest volume (mL)
**C1**	31.3	0	2.1	-980
**DF**	31.3	31.3	2.1	-1049
**C2**	31.3	0	2.1	-491

All flow rates and volumes of the individual operations steps are listed. – represents the withdrawal of liquid through the TFDF membrane. C1: concentration 1, DF: diafiltration, C2: concentration 2. Medium was used for the DF step.

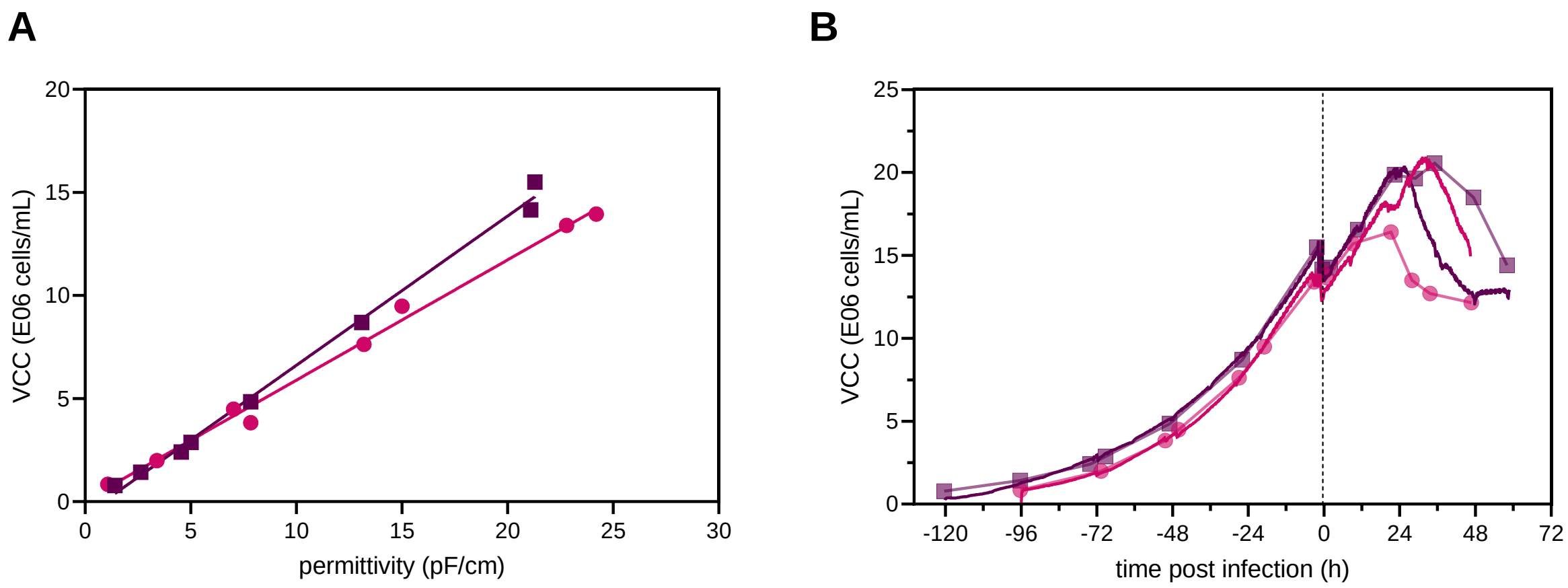

Fig. S1 A Correlation of VCC with permittivity signal during cell growth for TFDF1 (purple squares) and TFDF2 (pink circle). The slope of the linear regressions corresponds to 0.72 and 0.6, respectively. **B** Off-line VCC measurements (symbols) compared to on-line measurements of VCC (solid lines) for both replicates. Post infection, the on-line measurements started to deviate from the off-line VCC measurements.

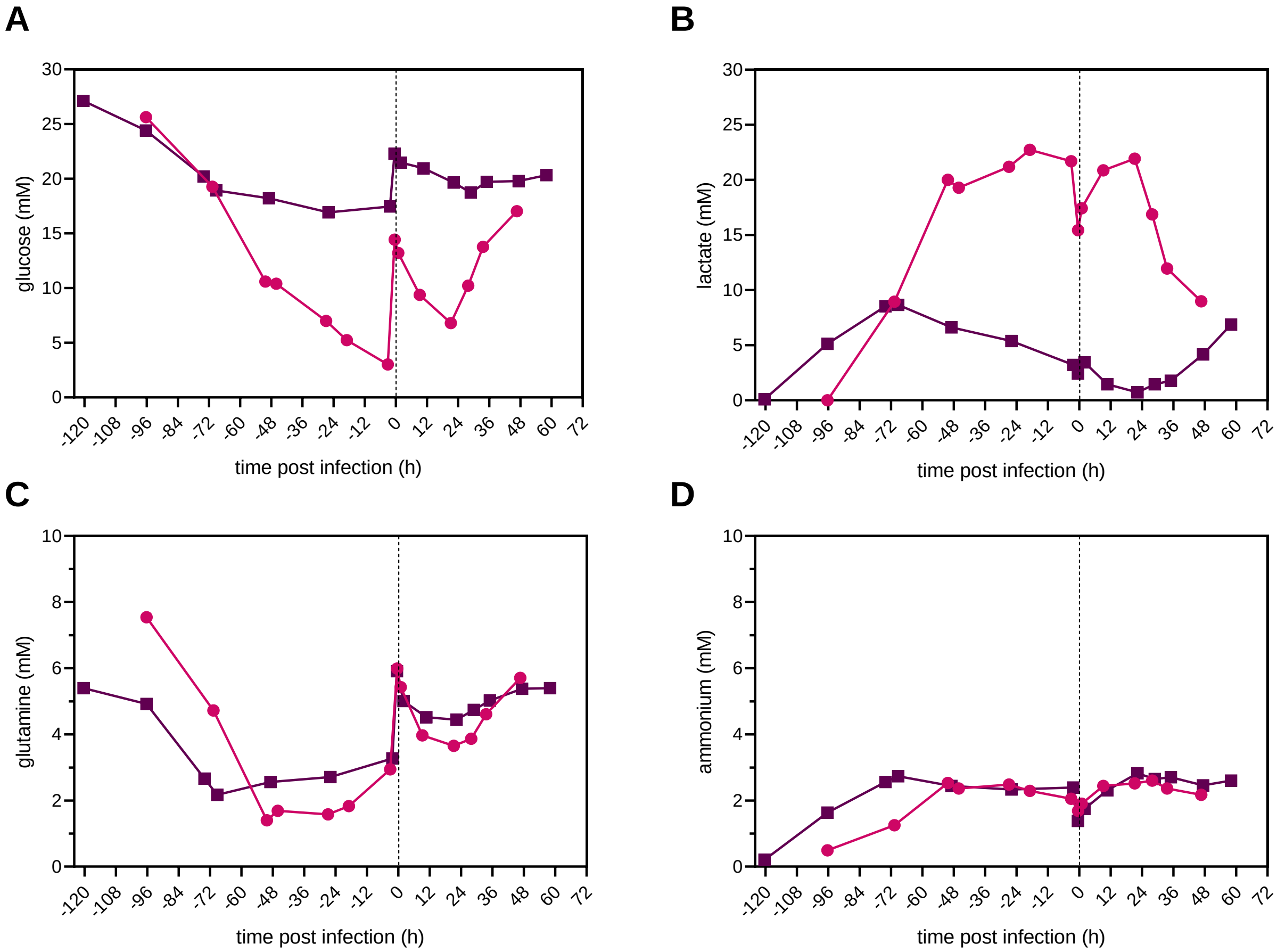

Fig. S2 Metabolite profile for TFDF runs with BHK-21 cells. The first TFDF run (TFDF1, purple squares) was adjusted manually, while the second TFDF run (TFDF2, pink circles) was controlled based on biovolume measured by a capacitance probe. A) glucose, B) lactate, C) glutamine, D) ammonium concentrations in cell culture supernatants were measured with a Cedex Bio Analyzer (Roche, Switzerland). Dashed line indicates that a RV with fresh medium was exchanged prior to infection at an MOI of 1E-4 and that temperature was lowered from 37°C to 34°C.

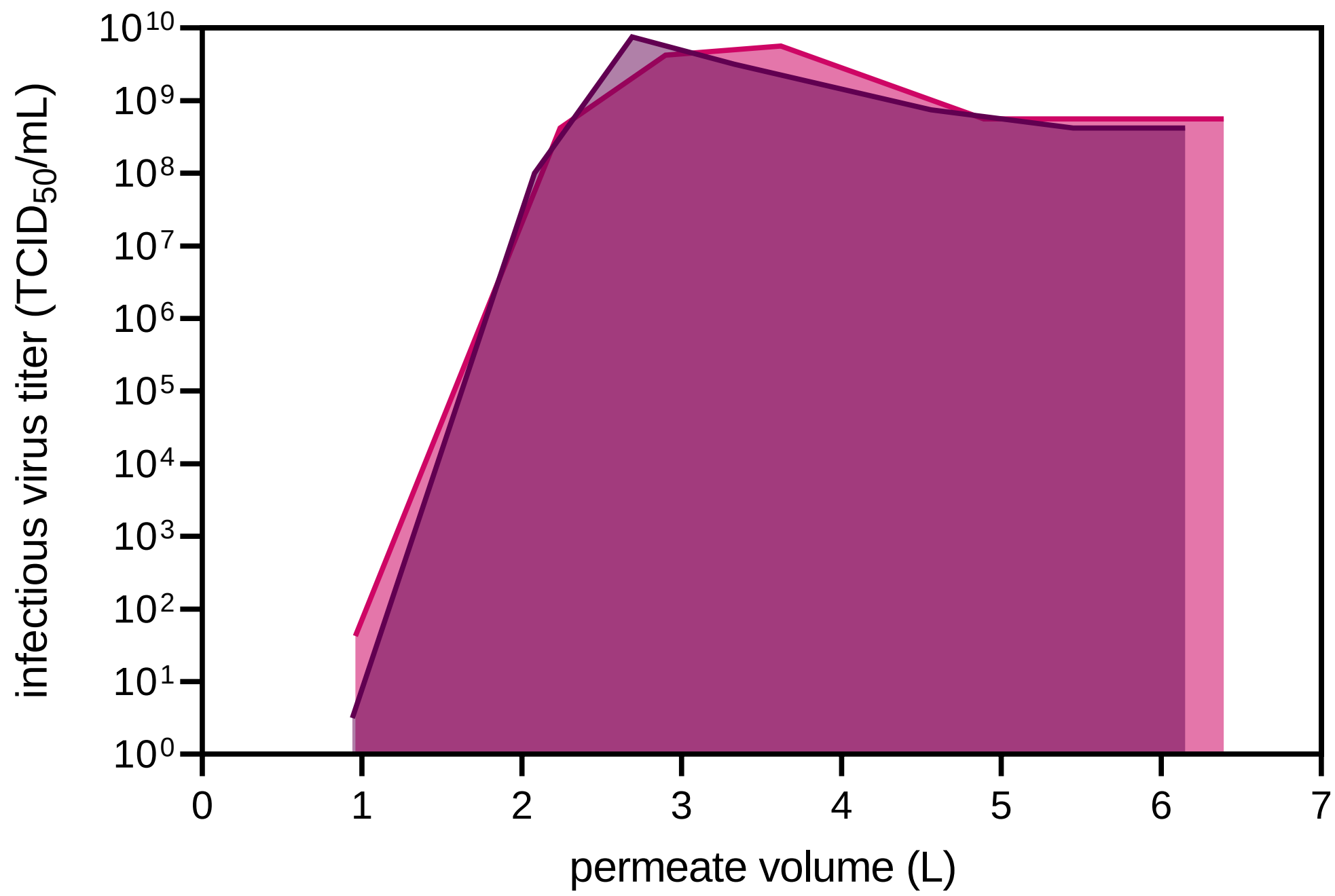

Fig. S3 Total rVSV-NDV production in TFDF1 (purple) and TFDF2 (pink). Infectious virus titer is plotted against the permeate volume. The area under the curve represents the maximum number of available infectious rVSV-NDV particles collected. A maximum amount of $9.0x10^{12}$ $TCID_{50}$ and $1.0x10^{13}$ $TCID_{50}$ are estimated for TFDF1 and TFDF2 harvesting, respectively.

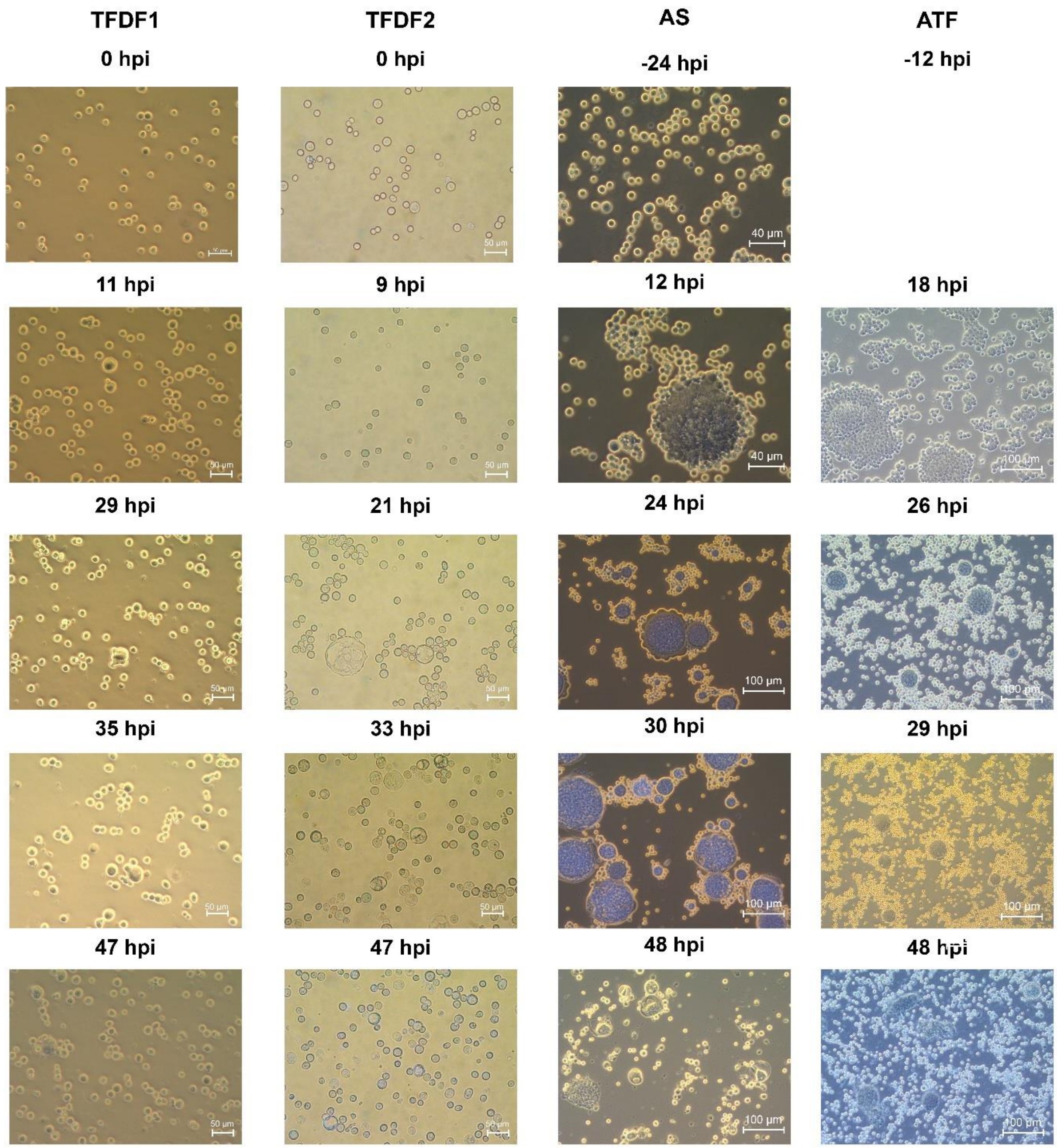

Fig. S4 Syncytia formation during rVSV-NDV production in perfusion cultures using TFDF, ATF, or acoustic settler for cell retention. Compared to previous acoustic settler cultivations (Göbel et al. 2023), where clusters of enlarged and fused cells appeared after 12 hpi, and the majority of the cell population was part of such a cluster, only sporadic syncytia formation was observed for both TFDF cultures. For ATF cultures, syncytia formation was more pronounced than for TFDF cultures. TFDF: tangential flow depth filtration; ATF: alternating tangential flow filtration; AS: acoustic settler.

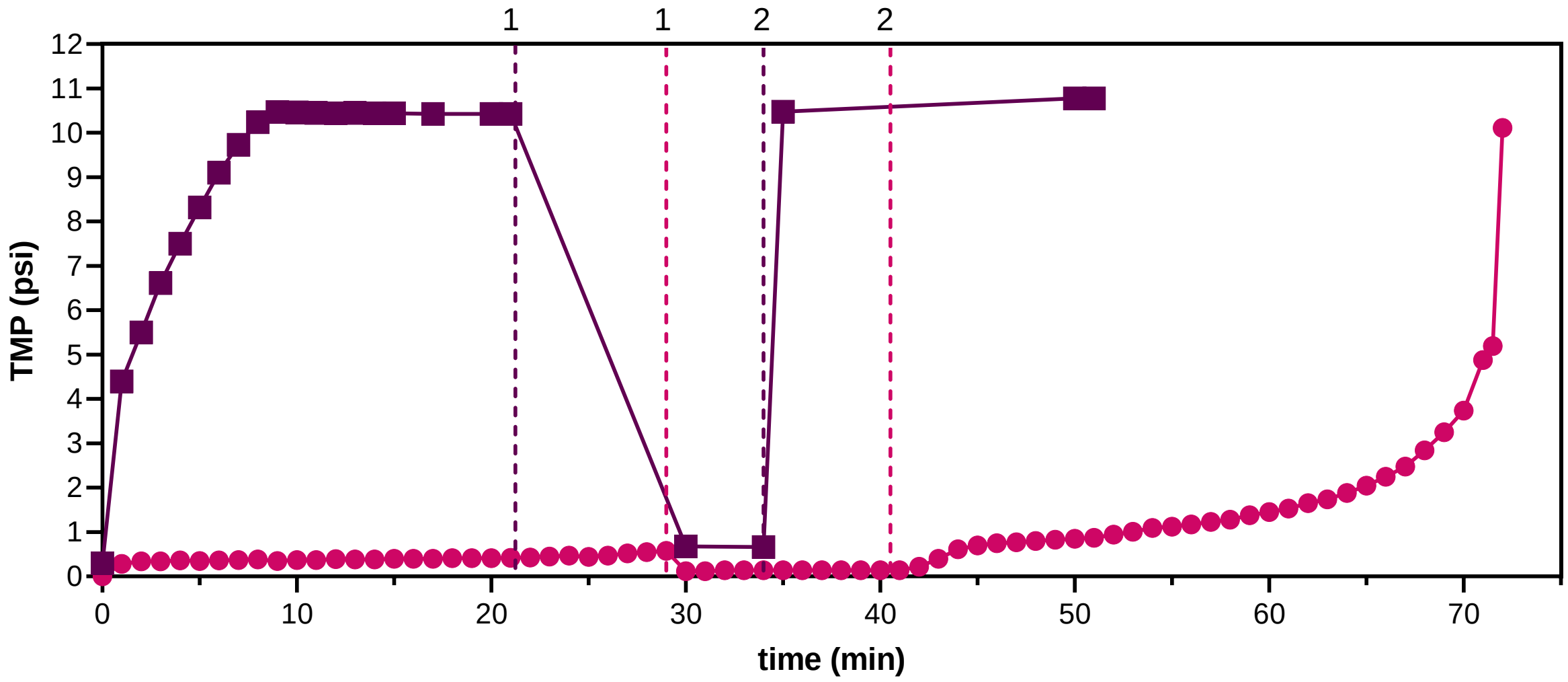

Fig. S5 Transmembrane pressure (TMP) profile during final reactor harvesting. TFDF1 (purple squares) and TFDF2 (pink circles) used a C1/DF/C2 process, where the diafiltration was carried out either using sterile PBS (TFDF1) or sterile supplemented medium (TFDF2). Dashed lines indicate the switch from initial concentration to diafiltration (1) and diafiltration to final concentration (2).

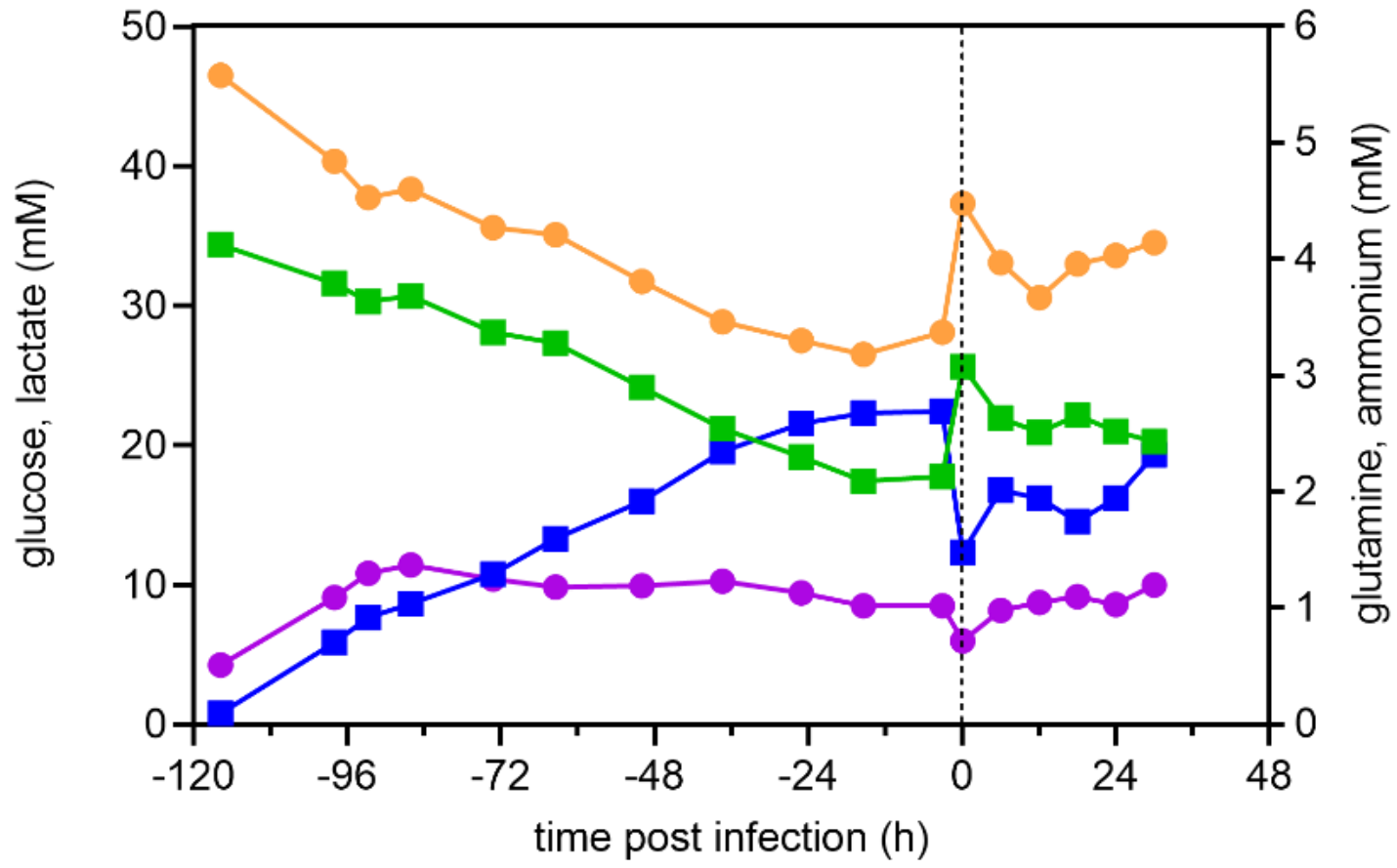

Fig. S6 Metabolite concentrations for rVSV-GFP production in HEK293-SF cells in perfusion mode using a 3 L STR coupled to a TFDF system. Glucose (green squares), lactate (blue squares), glutamine (orange circles), ammonium (purple circles) were measured with a Bioprofile FLEX 2 (Nova Biomedical, USA). Dashed line indicates that a RV with fresh medium was exchanged prior to infection at an MOI of 1E-3 and that temperature was lowered from 37°C to 34°C.

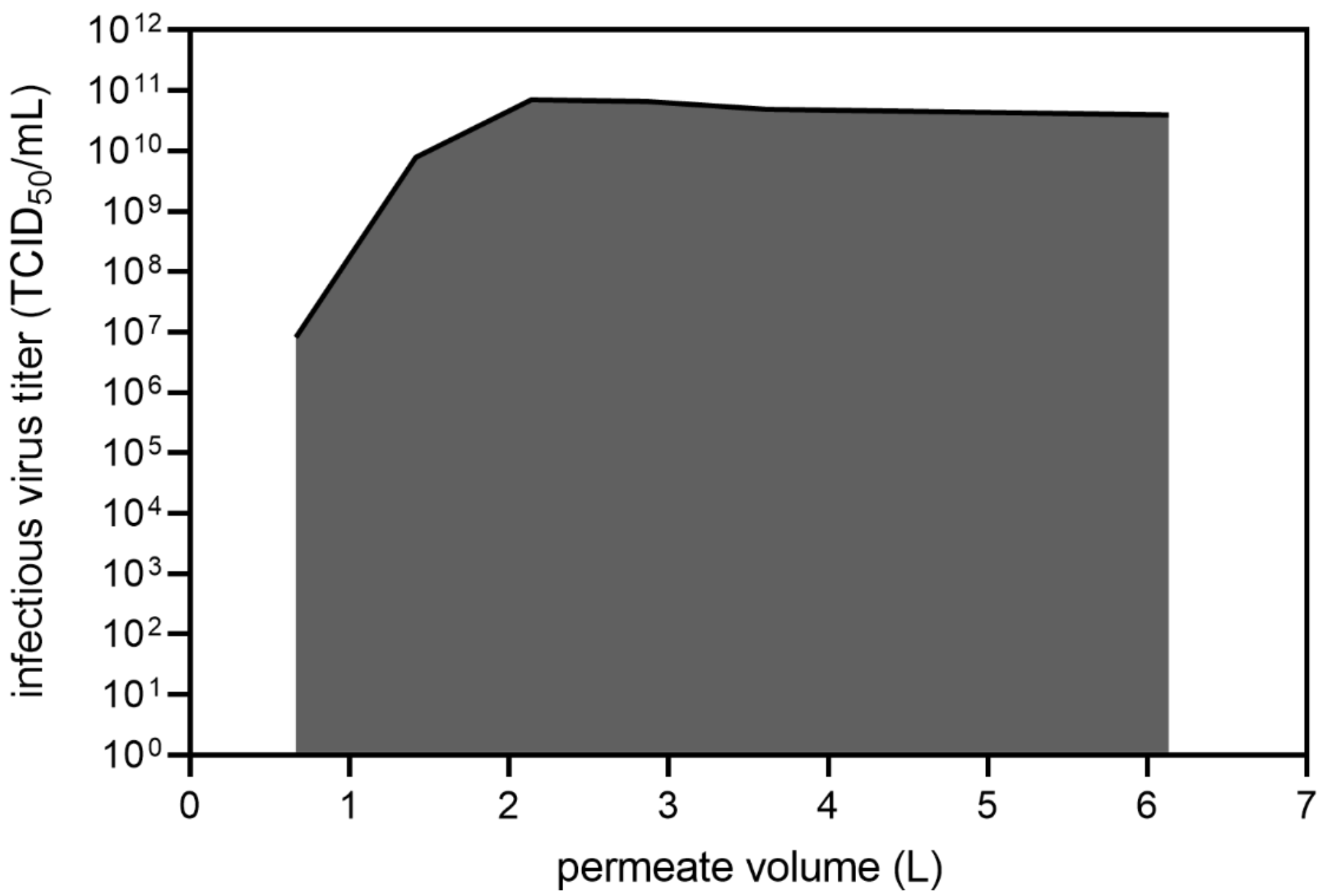

Fig. S7 Total rVSV-GFP production in perfusion mode using TFDF. Infectious virus titer is plotted against the permeate volume. The area under the curve represents the maximum number of available infectious rVSV-GFP particles collected. A maximum amount of 2.4×10^{14} $TCID_{50}$ is estimated.
